# Ecofriendly amino acid-tailored Fe/Ni nanomagnets for efficient adsorptive removal of food azo dyes (E123/E151) *via* electrostatic–hydrophobic contacts

**DOI:** 10.1039/d5na01180a

**Published:** 2026-08-04

**Authors:** Qurrat-ul-Ain, Abira Saleem, Muhammad Raza Shah, Muhammad Imran Malik

**Affiliations:** a Department of Chemistry, University of Karachi Karachi 75270 Pakistan qurrat_chem@uok.edu.pk; b H. E. J. Research Institute of Chemistry, International Center for Chemical and Biological Sciences, University of Karachi Karachi 75270 Pakistan

## Abstract

Azo food dyes exceeding EFSA safety limits present substantial risks to public health and water ecosystems. Their persistent water contamination and treatment challenges (low uptake, slow kinetics, and bioincompatibility) necessitate efficient removal technologies aligned with sustainable development goals. Here, we explore the usefulness of l-histidine-functionalized zero-valent iron/nickel nanospheres (His@nZVINs) for rapid and efficient adsorptive removal of food azo dyes E123 and E151. The ferromagnetic, nanocrystalline His@nZVINs were synthesized *via* a simple, inexpensive route involving complexation and sequential Fe–Ni reduction. Adsorption performance and mechanism were systematically evaluated. For E123, removal efficiency (*R*%) was 99.7% attaining equilibrium in 15 min (at 0.5 g per L adsorbent dose, 20 µM dye concentration, and pH 4), while E151 showed slightly higher *R*% (99.9%) and slower equilibrium (20 min) under identical conditions. His@nZVINs favored smaller E123 for faster kinetics and more highly charged (tetra-anionic) E151 for higher *R*% (31.1–99.7% for E123; 49.0–99.9% for E151) under single-factor optimization. Zwitterionic His@nZVINs enhanced dye uptake under acidic conditions through electrostatic interactions, while hydrophobic (π–π) contacts and hydrogen bonding sustained high *R*(%) at the isoelectric point (97.6% for E123; 99.4% for E151). FT-IR spectra and effective dye desorption in NaOH or organic solvent mixtures validated these interaction mechanisms. Adsorption followed the Langmuir isotherm with maximal monolayer capacities of 122.0 mg g^−1^ (E123) and 131.6 mg g^−1^ (E151), outperforming many reported adsorbents. Kinetics obeyed a pseudo-2nd order model with dominant film diffusion, and thermodynamics indicated a spontaneous, exothermic physisorption process. MTT cytotoxicity and brine shrimp lethality assays verified adsorbent nontoxicity. Moreover, His@nZVINs exhibited excellent reusability and efficacy in electrolyte-rich real water matrices, highlighting their potential as a sustainable dye-remediation solution.

## Abstract 2

Efficient and sustainable aqueous-phase removal of toxic food colorants (E123/151) using ferromagnetic, noncytotoxic, and zwitterionic l-histidine-functionalized-Fe/Ni nanospheres, controlled by coulombic/π–π/steric forces.

## Graphical abstract

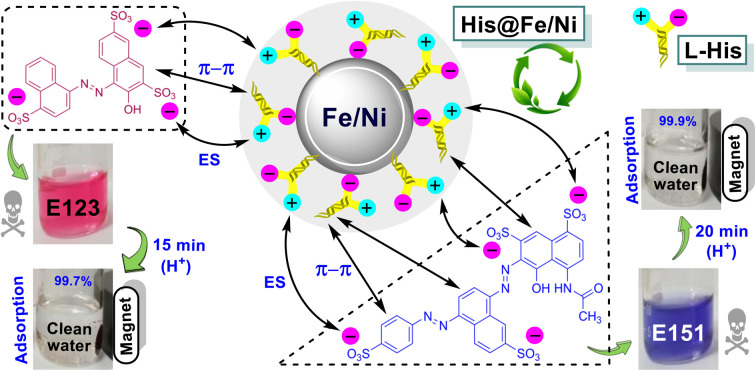

## Introduction

Clean water access is a primal human right, yet 2.1 billion people worldwide lack safe drinking water, while over 2 million die annually from water-borne diseases.^1^ Industrial agglomeration, while beneficial for the global economy, is a key contributor to water pollution.^2^ Industries, particularly food-processing, pharmaceuticals, textiles, cosmetics, and printing, massively discharge untreated or ineffectively treated colored wastewaters, heavily loaded with toxic dyes, heavy metals and other harmful chemicals.^3^ Synthetic azo (–N=N–) dyes account for over 60% of global dye production (∼7 × 10^5^ metric tons annually), and about 15–50% of these dyes may enter waterways due to weak substrate binding or inefficient dyeing techniques.^4^ Azo dyes, with complex aromatic structures, remain highly stable, recalcitrant, non-biodegradable, and resistant to conventional wastewater treatments.^5,6^ Their extreme stability and potential for aromatic amine production upon reductive cleavage render many azo dyes severely toxic, with recorded teratogenic, mutagenic, carcinogenic, and allergic effects in humans and wildlife.^4,7^ In addition, these persistent dyes, even in low amounts, can block sunlight, impair photosynthesis, deplete dissolved oxygen, and bioaccumulate in aquatic environments, raising serious ecological and public health concerns.^4^

**Fig. 1:**
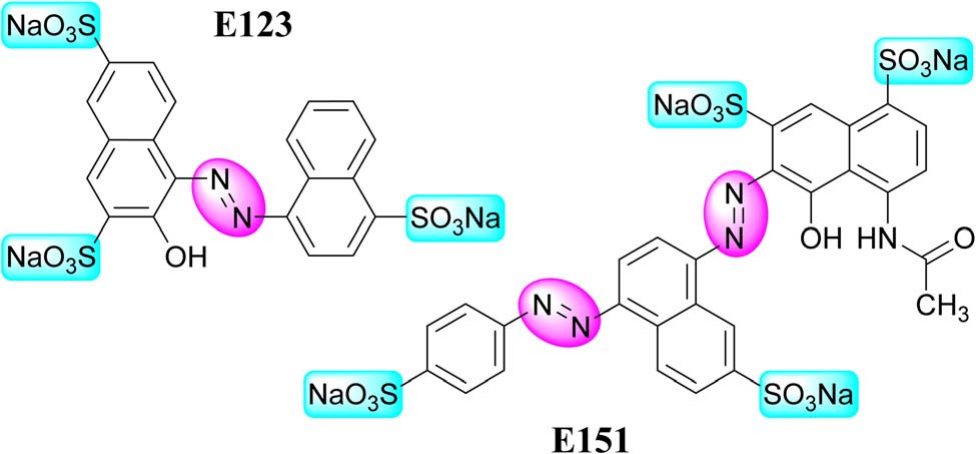
Molecular structures of two anionic food azo dyes employed in the present study.

Azo dyes constitute 65% of food additives for vibrant and appetizing coloration of multiple food products at low-cost, but with no nutritional value.^7^ Constant exposure to artificial food dyes above Acceptable Daily Intake (ADI) limits can lead to serious health issues, especially in children, including asthma, allergy, anxiety, attention-deficit/hyperactivity disorder (ADHD), cancer, and genotoxicity.^7,8^ Amaranth (E123) and Brilliant Black BN (E151), selected for this study (Fig. 1), are widely applied anionic azo food dyes of notable health concerns, with European Food Safety Authority (EFSA)-regulated ADI limits of 0.15 mg kg^−1^ and 5 mg kg^−1^, respectively.^9^ E123 is a dark red-purple monoazo trisulfonate dye often used to color foods and confectionery (*e.g.*, candies, jams, ketchup, and cake decor), beverages, pharmaceuticals, textiles (wool and silk), cosmetics, paper, paints, pulp, leather, wood, photographic materials, and phenol-formaldehyde resins.^10,11^ Prolonged consumption of E123 is associated with respiratory problems, tumors, allergies, birth flaws, and endocrine disruption in humans.^12^ E123 has also shown potential toxicological impact on human hemoglobin *in vitro*.^13^ Due to its adverse health effects, E123 has been banned by the U.S. FDA (Food & Drug Administration) and in many other regions.^7,14^ Similarly, bisazo E151 (tetrasulfonate dye) with violet-purple appearance has a wide use as a coloring agent in food coatings or decorations (desserts, jams, ice cream, dairy drinks, caviar, fish, meat, vegetables, fruits, and sauces)^15^ and in other industries (textile, cosmetic, paper, pulp, and leather).^16^ E151 use is forbidden in the USA and Japan,^8^ due to health risks including skin irritation, hives, redness, asthma and child ADHD.^17^ Despite regulations and food labeling, safety concerns still persist.^7^ Given the widespread discharge of these food azo dyes into water and their ecological risks, researching and utilizing effective color removal technologies is essential to reconcile industrial growth with environmental protection.

Several physical, chemical, and biological techniques are commonly employed to overcome dye pollution, but every method faces one or more limitations and challenges, including dye stability and diversity, complex wastewater composition, technology constraints, energy/resource costs, sludge/secondary-pollution/eco-safety, scalability, regulatory compliance, bioincompatibility, and low efficiency.^4,5^ Adsorption is rather simple, inexpensive, scalable, and accessible with various materials and is of great current interest as a feasible alternative for colored-wastewater treatment.^18,19^ However, traditional adsorbents, such as activated carbon, zeolites, clays, alumina, silica, and biochar,^18,20^ often suffer from low selectivity, limited reusability, slow kinetics, and reduced efficiency for complex or dilute contaminants,^19–21^ leading to a growing research demand for safe, sustainable, and efficient innovative adsorbents to advance color removal technology.

Metallic nanoparticles (NPs), particularly magnetic nano-zerovalent iron (nZVI)-based bimetallic NPs (BNPs) or nanoalloys (Fe/Ni, Fe/Pd, Fe/Zn, Fe/Cu, *etc.*), have recently gained attention as smart, responsive nanomaterials for wastewater treatment.^3,22,23^ Based on synergic mechanisms, nZVI-BNPs proffer enhanced adsorptive and catalytic dye removal, improved plasmonic, thermal, and magnetic properties, and additional advantages such as chemical stability, high surface area, no internal diffusion resistance, tunable size/shape and functionality, faster kinetics, and better reusability, compared to monometallic NPs.^6,24,25^ Challenges such as agglomeration, surface passivation, limited stability, low multitarget versatility, and environmental incompatibility of most monometallic or uncoated nanoadsorbents under practical conditions restrict their large-scale use.^26,27^ However, elemental iron is non-toxic and biocompatible, having high reductive efficiency (*e.g.*, for heavy metals and chlorinated organics) without generating toxic load, compared to other materials.^24^ Depositing a second metal (Ni, Cu, or noble metal) over nZVI to form BNPs preserves the Fe^0^ core, leading to its reduced agglomeration/corrosion and increased pollutant removal activity.^6^ Surface modification with organic coatings, such as polymers (polyvinyl alcohol and polyacrylic acid),^6^ biopolymers (carboxymethyl cellulose, chitosan, starch, and gum),^26^ ionic surfactants,^28^ or plant extracts,^29^ is another strategy to boost nZVI stability, biocompatibility, target specificity, and reactivity over prolonged periods. The use of amino acids such as glutamine, serine, alanine, and glutamic acid as eco-friendly mediators for double-shell nZVI formation (nZVI core, inner iron oxide/hydroxide shell, and outer organic layer) in water is also documented, with augmented pollutant removal reactivity and kinetics.^6,30^

Amino acids (AAs) are increasingly used in material modification due to their non-toxicity, availability, affordability, biocompatibility, size-control capability, structural stability, pH-responsiveness, and ability to provide ionic, hydrophobic, and hydrogen bonding interaction sites for surface connectivity.^30–32^ Iron-based magnetic materials modified with AA-ionic liquids,^33^ lysine,^34^ phenylalanine,^35^ glycine,^36^ and histidine^37^ are of significant importance for adsorbing toxic azo dyes including Congo red, Methyl Orange (MO), direct red 13, and acid black 1. l-Histidine (l-His), containing imidazole, –COOH and –NH_2_ functionalities, is a versatile AA stabilizer for numerous NPs. For example, l-His has been used to functionalize nanosized B/Fe doped graphene (for H_2_O_2_ detection),^38^ copper (Fe^3+^ sensing),^39^ silver (antioxidant and antibacterial),^40^ gold (streptomycin detection),^41^ ZnO (antibacterial),^42^ and Fe_3_O_4_ (Knoevenagel–Michael catalysis).^43^ However, the use of l-His to functionalize nZVI-based BNPs remains rarely explored.

Among iron-based BNPs, nZV Fe/Ni nanoparticles (nZVINs) have attracted considerable interest by virtue of their fascinating features, such as low cost, superparamagnetism, high stability, and fast turnover rates.^44^ nZVINs can act as potential catalysts^45^ or adsorbents for a wide range of dyes, including diazo direct red 16,^46^ monoazo orange G^47^ and MO,^48^ triphenylmethane dyes (malachite green and methylene blue),^49^ sulfonphthalein bromophenol blue,^50^ phthalocyanine direct blue 86,^51^ and indigo carmine (diagnostic, food-grade, and indicator dye).^52^ Most reported nZVINs are applied in bare form or modified with chitosan, but literature on their functionalization with AAs, specifically l-His, to advance food azo dye adsorption is very limited, highlighting a clear gap for further research.

For the selected food dyes, a variety of adsorbents other than nZVINs have been explored, such as polyelectrolyte-Fe_3_O_4_,^16^ iron-hydrochar from orange peel,^8^ kaolinitic clay,^53^ and chitosan–lignin–TiO_2_ (ref. 54) for E151, and pink clay,^14^ Fe_3_O_4_–MgO,^55^ potato-peel biosorbent,^11^ adipic acid–pectin–Fe^0^/Fe_3_O_4_,^56^ ZnO,^10^ and aminated avocado seed^57^ for E123. However, their reported adsorption capacities (*q*_max_ = 1.0–98.9 mg g^−1^) remain relatively low, emphasizing the need for better alternatives.

Recognizing the research gap and potential synergy in combining nZVINs and l-His, the present study is directed toward the synthesis, characterization and evaluation of l-His-modified nZVINs (His@nZVINs) as a novel adsorbent for removing E123 and E151 from aqueous media. Investigating various structural and performance parameters, along with isothermal, kinetic, and thermodynamic modeling, would provide deep insights into the dye removal mechanism driven by His@nZVINs. As signified by recent reviews on nZVI-based dye remediation^6^ and multifaceted BNPs toward sustainable development goals (SDGs),^23^ claims of an adsorbent's sustainability and economic viability for industry are incomplete without assessing its environmental footprint. Accordingly, this work also aimed to examine the reusability and toxicity of His@nZVINs as a suitable food dye adsorbent.

## Experimental

### Chemicals

The analytical-grade chemicals were used exactly as received, with no additional refinement. Amaranth (E123) and Brilliant Black BN (E151) dyes (Fig. 1) were provided by BDH Laboratory Supplies (UK) and Sigma-Aldrich (Germany), respectively. Iron and nickel metal salts (FeCl_3_·6H_2_O and NiCl_2_·6H_2_O), l-histidine (C_6_H_9_N_3_O_2_), and sodium hydroxide (NaOH) were procured through Merck (Germany), and sodium borohydride (NaBH_4_) was procured through Daejung (Korea). Honeywell (Germany) supplied acetic acid (CH_3_COOH), hydrochloric acid (HCl), and sodium chloride (NaCl), while Tedia Company, Inc. (USA) supplied ethanol and methanol. An ELGA Cartridge (Type C114) water demineralization system was used to generate deionized (DI) water from distilled water and applied after deoxygenation *via* a vacuum pump.

### Fabrication and structural design of His@nZVINs

l-His-modified Fe/Ni-BNPs (His@nZVINs) were synthesized *via* a simple, cost-effective, and environmentally safe wet chemical reduction method (Scheme S1), with some modification in the literature procedure.^49^ All the solutions were prepared in DI water, and the reaction was carried out in a 250 mL anaerobic three-necked round-bottom flask under argon gas supply. First, 43 mL of freshly prepared solution of FeCl_3_·6H_2_O (1.2 g) was added to 60 mL of 150 mM l-His solution. The resulting reddish-brown solution was agitated for 15 min. Then, 126 mL of NaBH_4_ (106 mM) solution was transferred drop-wise to the brown solution under vigorous stirring in an inert atmosphere leading to rapid production of black precipitates. The resulting mixture was agitated for 15 min after adding 6 mL of NiCl_2_·6H_2_O (0.3 g) solution. Finally, an external magnet was used to isolate the fine brown-black BNPs (His@nZVINs) from the mixture. To remove surplus borohydride or other unnecessary chemicals, the separated brown-black solid was rinsed thoroughly with DI water, rinsed with ethanol, and dried for 24 hours in a vacuum at room temperature. For comparison, bare nZVINs were also synthesized according to the same method, but without the addition of l-His.

The synthesis of His@nZVINs involved three main steps (Scheme S1). Excess l-His initially complexes with Fe^3+^ to form an l-His-Fe^3+^ intermediate. In the second step, sodium borohydride reduces Fe^3+^ to Fe^0^ following eqn (1) under an inert (argon) atmosphere, yielding l-His-stabilized Fe^0^-NPs (His@nZVIs).^45^ Finally, excess Fe^0^ reduces Ni^2+^ to Ni^0^, according to eqn (2).^49^ The final step is based on an electroless plating process, where Ni^0^ NPs are deposited on Fe^0^ NPs *via* chemical reduction in the presence of l-His to form His@nZVINs. The applied molar ratio of reagents (Ni^2+^/Fe^3+^/NaBH_4_) for synthesis was kept at 1 : 3.5 : 10.5 considering reaction stoichiometry. A continuous circulation of argon gas was essentially maintained to prevent metal oxidation during synthesis.

1 4Fe^3+^ + 3BH_4_^−^ + 9H_2_O → 4Fe^0^ ↓ + 3H_2_BO_3_^−^ + 12H^+^ + 6H_2_↑

2 Fe^0^ + Ni^2+^ → Ni^0^ ↓ + Fe^2+^

### Characterization

Surface functional groups and bond types in synthesized BNPs were identified using Fourier transform infrared spectra, recorded at 400–4000 cm^−1^ on KBr pellets at room temperature employing an FT-IR spectrometer, model Shimadzu IR-460. The particle surface morphology and dimensions were investigated through scanning electron microscopy (SEM) with instrument model JSM-6380A, Jeol, Japan; suitably magnified SEM images were captured at an operating accelerated voltage of 20 kV upon loading of powdered samples onto carbon-coated copper grids. Energy-dispersive X-ray spectroscopy (EDX; X-MaxN 80, Oxford Instruments) coupled with SEM (Tescan Mira 3) was employed to determine the elemental composition and verify surface functionalization of the BNPs. The BNP size analysis was also performed by atomic force microscopy (AFM), instrument model Agilent 5500, using silicon cantilevers with a force constant of 0.02–0.77 N m^−1^ and 10–15 micron tip height in contact mode. The thermal properties of BNPs (5–10 mg powder) were examined using a thermogravimetric analyzer (TGA, SDT-Q600 Version 8.3 Build 101) over 50–990 °C at a 10 °C per minute heating rate in an N_2_ atmosphere. The diffraction patterns for crystallinity and related characteristics of BNPs were obtained from XRD (X-ray diffractometer model PANalytical X'Pert Pro), using a copper-Ka radiation source, at 30 mA current, and at an accelerating voltage of 40 kV. Ambient-temperature magnetic characteristics of BNPs were determined *via* a vibrating sample magnetometer (VSM, model FCM-10). The zeta potential (at pH 2–14) for determining zero-point charge (pH_PZC_) on the surface of BNPs was measured using Malvern (UK) Zetasizer Nano ZS90 equipment, using a 1 g per L sample. The absorbance of dye solutions was recorded using a BK-UV1800 PC (Biobase) UV-visible spectrometer in a quartz cuvette (10 mm path length). For measuring pH of solutions, a HANNA pH meter, model H12211, was used. The separation and recovery of magnetic BNPs from the solution was accomplished using a simple kitchen magnet. Different real water matrices were characterized using an ADWA TDS/EC meter (AD330), a Lovibond SD 90 salinity meter, a Jenway PFP7 flame photometer (Na^+^/K^+^) and standard water/wastewater examination methods, including titration for Ca^2+^, Mg^2+^, Cl^−^, and HCO_3_^−^, spectrophotometry for NO_3_^−^, and gravimetry for SO_4_^2−^.^58^

### Batch adsorption experiments

For comparative pilot (unoptimized) adsorption studies of E123 and E151 dyes in batch mode, 0.5 g per L His@nZVINs was introduced into 25 mL dye solution (20 µM) in DI water at ambient pH (∼6.7) and temperature (25 °C). The reaction mixture was agitated using a thermostatic mechanical shaker (SWB-A, BIOBASE) at 200 rpm speed until equilibrium (no further dye removal) was attained. The His@nZVINs were then magnetically isolated from the unbound dye solution, as depicted in some real photos in Fig. S1. The residual dye concentrations in supernatant solutions were estimated from spectrophotometric standard calibration curves using absorbance measured at 514 nm (E123) or 566 nm (E151). For optimized dye removal using His@nZVINs, single-factor batch adsorption studies were conducted by systemic variation of target parameters (pH: 2–13, salt: 0–1 M NaCl, temperature: 25–85 °C, adsorbent dose: 0.5–4.5 g L^−1^, and initial dye concentration: 11.7–60.3 mg L^−1^ for E123 and 17.3–60.7 mg L^−1^ for E151) while keeping all other parameters constant. For pH adjustments, 1 M HCl or NaOH solution was used. The factors fixed for optimization studies were a temperature of 25 °C, adsorbent dosage of 0.5 g L^−1^, pH of 4 (for dye concentration study) or ambient (for other studies), and dye concentration of 25 µM (for dose study) or 20 µM (for other studies). The impact of time on dye adsorption was evaluated for almost all above sets of experiments, and adsorption equilibrium time (*t*_eq_) needed for saturation was also determined against each target variable for comparison. The adsorption of dyes in four different real water specimens (drinking water, tap water, groundwater, and seawater) was also tested and compared with adsorption data from DI water at two different initial dye concentrations (20 µM and 50 µM) at ambient pH and temperature, while retaining the adsorbent amount constant at 0.5 g L^−1^. These real water samples were gathered from various sources: the drinking water sample from an RO-plant at Gulistan-e-Johar (Block-7), tap water from the laboratory of Chemistry (U.O.K.), groundwater from the Korangi Industrial Area, and seawater from Paradise Beach Point in Karachi.

The adsorptive capacity (*q*) in mg g^−1^ and percent dye removal efficiency (*R*%) of His@nZVINs at various stirring intervals was calculated using eqn (3) and (4), respectively.3
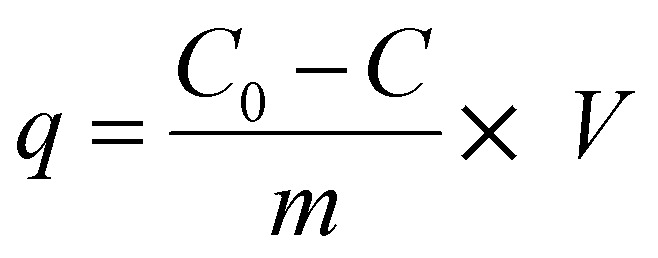
4
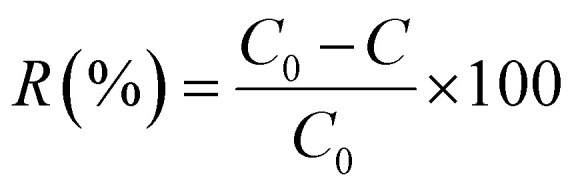
where *m* represents the dry mass (g) of adsorbent His@nZVINs, whereas *V* represents solution volume (L) of the dye. The initial dye concentration in solution at *t* = 0 min is represented by *C*_0_, while the dye concentration in the supernatant at certain time (*t*) is represented by *C* (mg L^−1^). For equilibrium data, *q*_e_ substitutes *q* and *C*_e_ substitutes *C* in eqn (3), where *q*_e_ and *C*_e_ denote the equilibrium adsorptive capacity (mg g^−1^) of His@nZVINs and the equilibrium dye concentration in the liquid (mg L^−1^), correspondingly. Each set of experiments was run in triplicate to guarantee result reproducibility, and arithmetic mean values within ±5% maximum standard deviation were reported.

To gain mechanistic insight into dynamic adsorbate–adsorbent interactions at equilibrium, particularly the process of transferring dye molecules from bulk liquid to the adsorbent, four isothermal models (Langmuir, Freundlich, Dubinin–Radushkevich, and Temkin)^59^ were analyzed for best fitting of equilibrium adsorption data from varying dye concentration study. The parameters for each isothermal model, generalized with a straight-line expression (Table S1), were computed from respective plots to get crucial details about the adsorption mechanism and adsorbent surface characteristics. For a comprehensive understanding of the adsorption rate and diffusion mechanisms, the adsorption data at varying time intervals under certain conditions of His@nZVINs dose (1.5 g L^−1^), initial dye concentration (25 µM), and temperature (25 °C) were applied to five kinetic models (pseudo-1st order, pseudo-2nd order, Elovich, intraparticle diffusion, and Boyd), in the form of plots using respective linearized mathematical relationships (eqn (S1)–(S5)).^16,29^ The most appropriate model is selected based on the highest correlation coefficient (*R*^2^), which measures how closely a model matches the experimental data. Evaluating thermodynamics is another pivotal facet in investigating adsorption mechanisms, offering a comprehensive insight into the impact of temperature on adsorption mode. Thermodynamic variables, *i.e.*, the variation in standard entropy (Δ*S*°), Gibbs free energy (Δ*G*°), and enthalpy (Δ*H*°), were calculated based on the adsorption data from study of temperature (varying at 10 K intervals from 298 K to 358 K).^46^

### Batch desorption and reusability

To regenerate used His@nZVINs after adsorption, a 9 : 1 (v/v) CH_3_OH–CH_3_COOH mixture, 1 M NaOH, and 1 M HCl were utilized as comparative desorption media. The used and dried His@nZVINs sample, with a certain amount of adsorbed dye, was treated with 25 mL desorbing eluent under ambient conditions and continued to stir for 65 min to attain maximum desorption. The percent dye desorption was calculated by measuring the relative rise in supernatant absorbance at dye *λ*_max_ (514 nm for E123 and 566 nm for E151) compared to the adsorbed dye. Each desorption experiment was executed in triplicate for data consistency. The revived adsorbent after desorption from the CH_3_OH–CH_3_COOH mixture and NaOH was thoroughly washed with distilled water, dried and reused up to five continuous adsorption–desorption cycles (under conditions identical to those of the first cycle) to test the reusability of His@nZVINs.

### Toxicity assays

The toxicity of His@nZVINs, together with its precursors and standard drugs for comparison, was evaluated using MTT cytotoxicity and brine shrimp lethality assays, as described below.

#### MTT assay

The *in vitro* spectrophotometric MTT [3-(4,5-dimethylthiazole-2-yl)-2,5-diphenyl-tetrazolium bromide] assay^60^ was conducted using two cell lines, 3T3 (healthy mouse fibroblasts) and HeLa cells (human cervical cancer). The cell culturing was performed in Dulbecco's modified Eagle's medium (3T3) or Eagle's minimum essential medium (HeLa), accompanied by streptomycin (100 mg mL^−1^), fetal bovine serum (5%), l-glutamine (2 mM), and penicillin (100 IU mL^−1^) under 5% CO_2_ (37 °C). The sample suspensions of 200 µL at 30 µg mL^−1^ were added to overnight-incubated cells (5 × 10^4^ cells per well) in 96-well plates. After 48 h of incubation, 200 µL MTT reagent (0.5 mg mL^−1^) was introduced and incubated further for four hours. The resultant blue formazan crystals were treated with 100 µL DMSO to dissolve and quantified by measuring absorbance at 550 nm. Percent cell growth inhibition was computed from comparison of absorbance of triplicate test samples with that of negative and positive control groups.

#### Brine shrimp lethality assay

Brine shrimp lethality is a rapid, inexpensive, and accessible bioassay to test sample ecotoxicity.^61^ For hatching and maturation as nauplii, 50 mg brine shrimp (*Artemia salina*) eggs were sprinkled into a hatching tray, containing filtered brine solution (38 g per L sea salt in water, pH 7.4) and incubated at 37 °C for 2 days under good aeration and illumination. Different volumes of stock sample (20 mg per 2 mL) for three varying concentrations (1, 10, and 100 µg mL^−1^) were transferred to vials (3vials/concentration), along with 30 larvae (shrimps) to each vial using a Pasteur pipette and volume was made to 5 mL with artificial seawater. After 24-hour incubation at 25–27 °C under illumination, live larvae were counted and % mortality was calculated. The reference cytotoxic drug and solvent (DMSO in sterile seawater) served as positive and negative controls, respectively.

## Results and discussion

### Characterization

The synthesized His@nZVINs exhibit a hierarchical hybrid structure comprising an Fe^0^-rich core, a thin Ni shell, and an outer l-His functional layer coordinated *via* O- and N-donor groups, providing enhanced dispersion, surface functionality, and multiple adsorption-active sites.

#### Bonding and surface functionalization

**Fig. 2:**
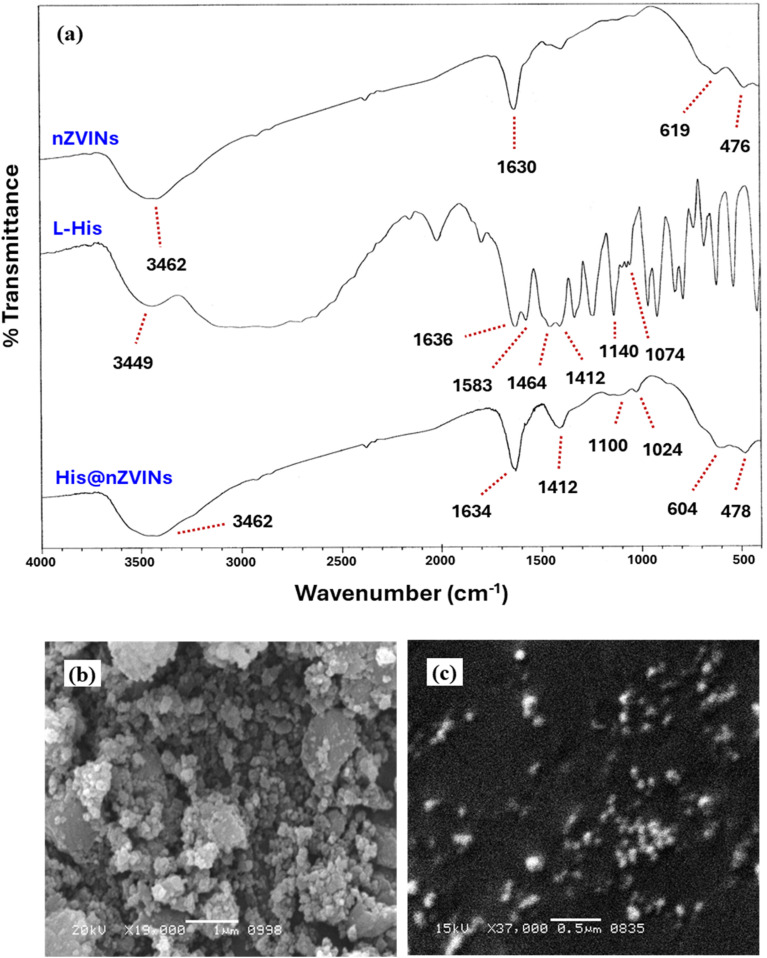
(a) FT-IR spectral comparison of bare nZVINs, l-His, and His@nZVINs. (b) SEM micrograph of bare nZVINs. (c) SEM micrograph of His@nZVINs.

FT-IR spectra (Fig. 2a) confirmed successful functionalization of nZVINs with l-His. The metal–oxygen vibration bands at 478 and 604 cm^−1^, along with a 15 cm^−1^ shift in the Fe–O band (619 → 604 cm^−1^) upon modification, provided evidence of coordination between metal centers and l-His oxygen-containing groups. The broad band at 3462 cm^−1^, attributing to overlapping N–H and O–H stretching vibrations, confirmed the presence of amine and hydroxyl functionalities on the His@nZVINs surface. Characteristic carboxylate (COO^−^) bands at 1412 and 1634 cm^−1^ (overlapping with amine bending), together with band broadening or shifting relative to pure l-His, indicated l-His adsorption (surface complexation) on Fe/Ni *via* carboxyl and amine groups in His@nZVINs. Furthermore, attenuation of imidazole C–N/N–H bands (1020–1160 cm^−1^) in His@nZVINs, compared with free l-His, indicated participation of the heterocyclic ring in binding to nZVINs.

These spectral changes confirm l-His anchoring to the Fe/Ni surface *via* multiple oxygen- and nitrogen-donor coordination sites, creating a multifunctional surface enriched with metal, carboxyl, amine, hydroxyl, and imidazole groups for enhanced dye adsorptive performance through electrostatic, hydrogen bonding, π-related, and dye–metal coordination interactions.

#### Morphology and size

**Fig. 3:**
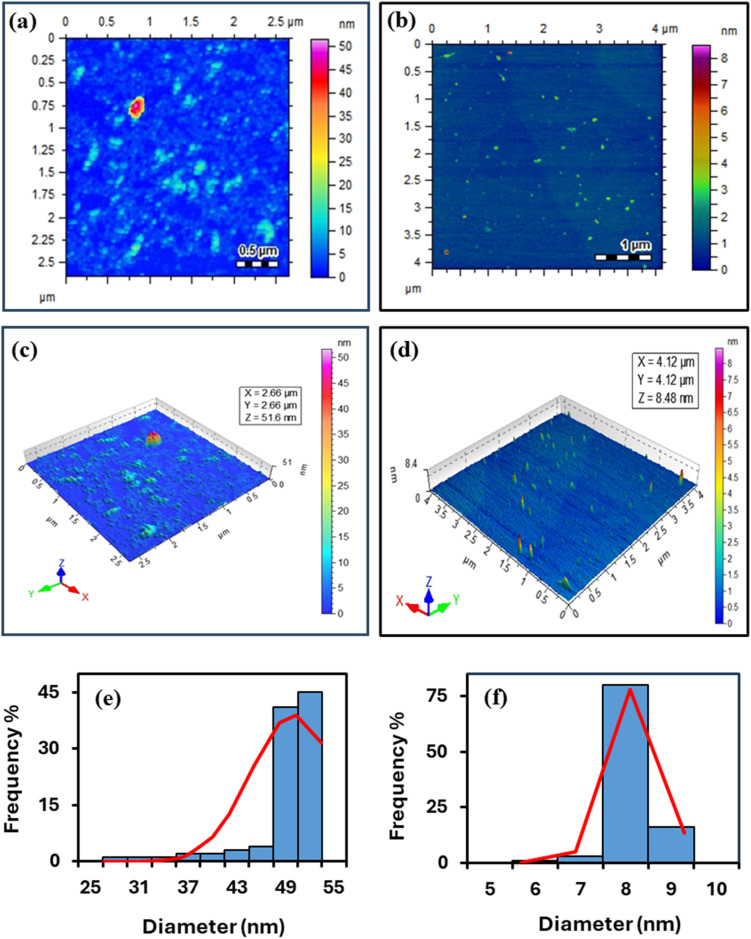
AFM study of His@nZVINs (RHS) and uncoated nZVINs (LHS). (a and b) 2D topography visuals, (c and d) 3D topography visuals, and (e and f) histograms for BNP size distribution.

Morphological analysis using SEM (Fig. 2b and c) and AFM (Fig. 3) revealed that both bare and coated BNPs exhibit quasi-spherical shapes. Bare nZVINs (Fig. 2b) formed polydisperse, chain-like aggregates (96–142 nm; average = 115 nm) due to magnetic interactions, whereas His@nZVINs (Fig. 2c) showed reduced aggregation and improved dispersion with a narrower size range (65–89 nm; average = 77 nm) in SEM images. AFM analysis (Fig. 3) further confirmed substantial size reduction after functionalization, showing primary particle sizes of 6–9 nm (average = 8 nm) for His@nZVINs and 26–52 nm (average = 49 nm) for uncoated nZVINs.

The discrepancy between SEM and AFM sizes likely stems from differing measurement principles, lack of cross-calibration, and variation in sampled particle populations. AFM (performed after sonication and controlled deposition) measures the vertical height of mostly individual nanoparticles, whereas SEM captures lateral dimensions of agglomerated particles in the dried state. Accordingly, SEM yields larger sizes due to aggregation, while AFM better represents primary particle dimensions. Thus, the discrepancy reflects primary particle size (AFM) *versus* aggregate size (SEM), with AFM providing more accurate estimates of the smallest nanoclusters.^62^

Overall, these results indicate that l-His acts as an effective capping and stabilizing agent, limiting magnetic agglomeration and promoting smaller, well-dispersed BNPs with improved surface characteristics.^63^

#### Elemental composition

**Fig. 4:**
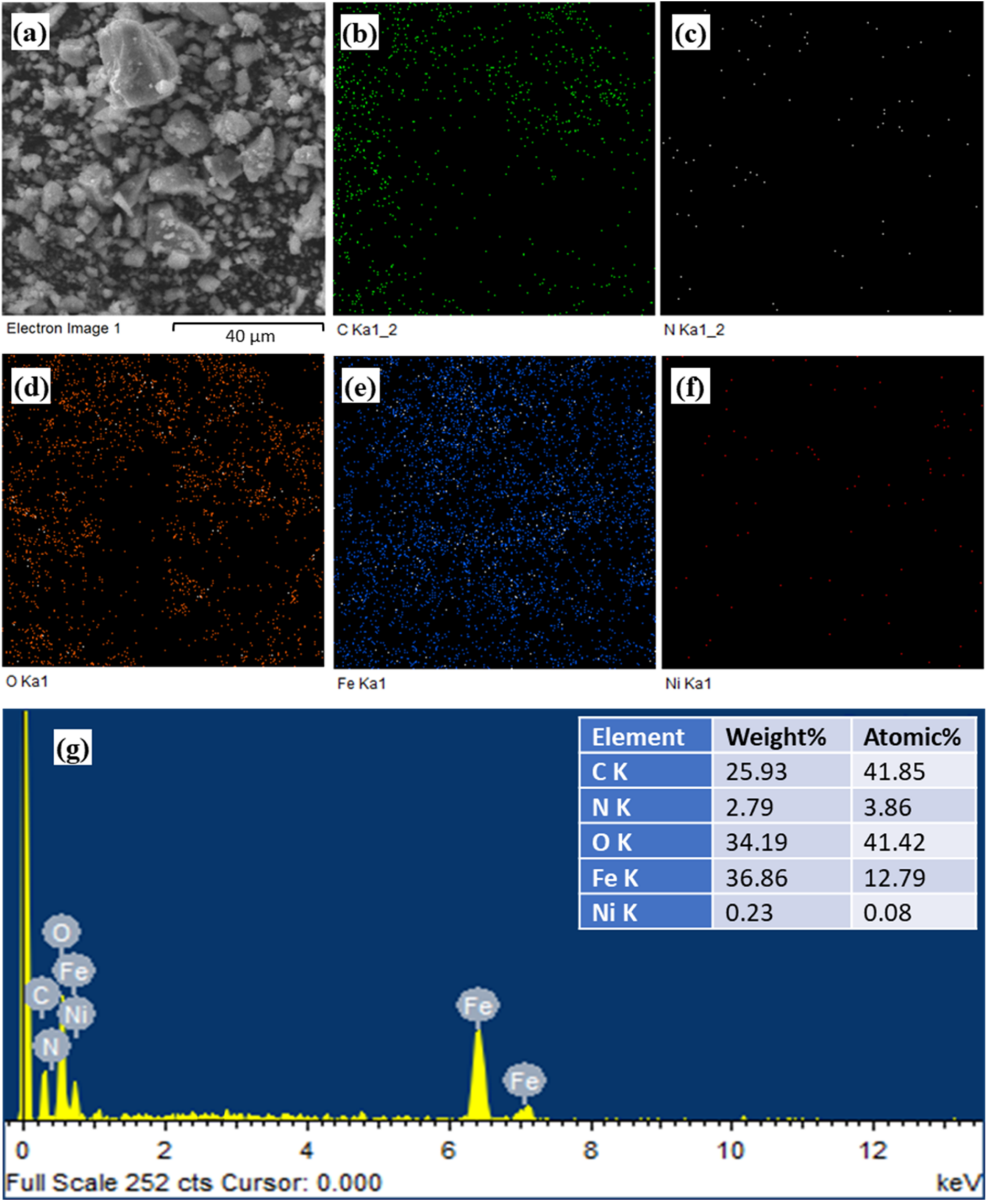
EDS analysis of His@nZVINs. (a) SEM microstructure of selected mapping area. (b–f) Corresponding EDS elemental mapping of C, N, O, Fe, and Ni, respectively. (g) EDX spectrum with elemental composition.

The SEM-EDX analysis (Fig. 4) confirmed the elemental composition of His@nZVINs, revealing the uniform distribution of C(25.9 wt%), N (2.8 wt%), O (34.2 wt%), Fe (36.9 wt%), and Ni (0.23 wt%). Notably, the presence of nitrogen, which was absent in the EDS spectrum of bare nZVINs (Fig. S2), provided direct evidence of nitrogen-containing functionalities introduced by l-His. Increased carbon and oxygen signals further verified the organic coating layer and oxygen-containing functional groups derived from l-His.

The Ni/Fe molar ratio in His@nZVINs (0.01, Ni_0.1_Fe_10_) was markedly lower than that in bare nZVINs (0.5, Ni_5_Fe_10_), which retained near-theoretical Ni content (Ni_4_Fe_10_). This reduction in Ni content suggests its partial leaching during functionalization, likely driven by the formation of a stable, water-soluble l-His-Ni^2+^ complex. Consequently, an Fe^0^-dominated core with a thinner Ni shell was formed, potentially enhancing surface reactivity and accessibility of active iron sites for dye adsorption.

#### Thermal stability and organic content

**Fig. 5:**
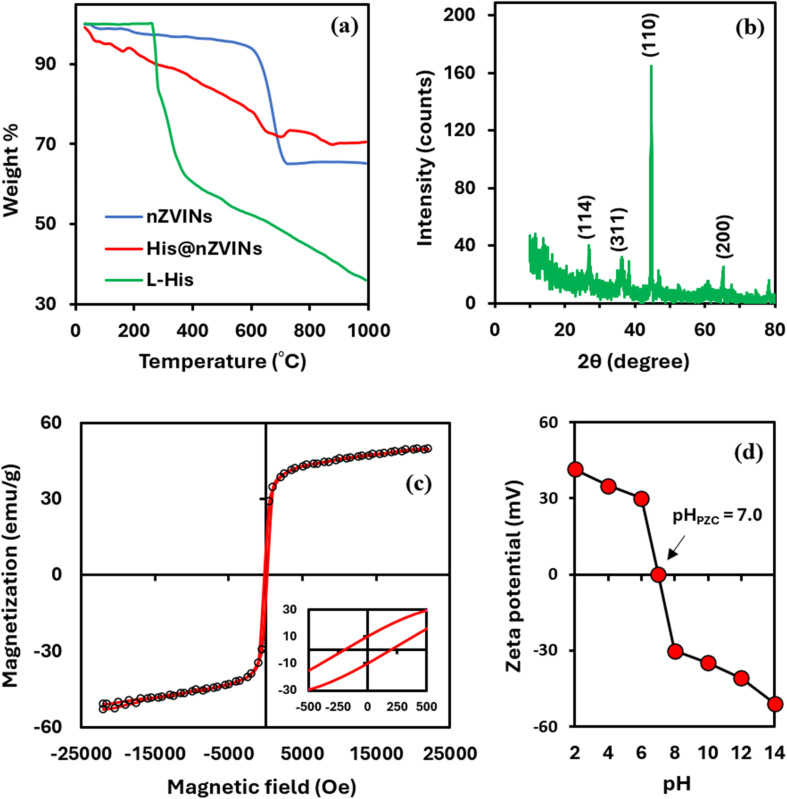
(a) TGA curves of His@nZVINs and precursors (pure l-His and bare nZVINs). (b) XRD pattern of His@nZVINs. (c) Magnetic hysteresis (*M*–*H*) loop of His@nZVINs; the inset shows the low magnetic field data of the *M*–*H* loop. (d) Point of zero charge determination for His@nZVINs.

TGA was applied to evaluate thermal behavior and to confirm and quantify organic loading on the nZVIN surface. As shown in Fig. 5a, His@nZVINs exhibited about 3.5 times higher weight loss (21.8%) compared to bare nZVINs within 30–600 °C, confirming organic l-His coating. The initial 4.9% weight loss up to 100 °C accounted for the release of surface adsorbed water, while subsequent loss corresponded to decomposition of coated l-His. Degradation of His@nZVINs almost stopped after 860 °C, with a total weight loss of about 29%. Minor weight gains near 180 and 800 °C may result from sample oxidation, possibly due to oxygen released from adsorbed water or the l-His carboxyl group in the closed thermal system. Notably, l-His on His@nZVINs started degrading at lower temperatures than pure l-His, likely due to catalytic effects of nZVINs that accelerate l-His thermal decomposition.^37^

Quantifying amino acid coating on Fe/Ni BNPs reveals the extent of surface functionalization and available active sites, while also reflecting stabilization against aggregation/corrosion, which directly influence dye adsorption performance. To determine the minimum l-His loading [%*W*_(His)_], mass data at 600 °C were used, as weight loss beyond this point for bare nZVINs showed irregular behavior (a sharp decline likely from impurity decomposition or release of strongly bound paramagnetic oxygen). The %*W*_(His)_ was calculated using eqn (5) by subtracting the weight loss (%WL) of bare nZVINs (6.1%) from that of His@nZVINs (21.8%) at 600 °C and correcting for adsorbed water losses (2.6% for bare and 4.9% for coated samples). The minimum %*W*_(His)_ was thus estimated to be 8.2%, confirming substantial surface functionalization.

5 %*W*_(His)_ = %WL_(__His@nZVINs__)_ − [%WL_(nZVINs)_ + %WL_(H_2_O,__His@nZVINs__)_ + %WL_(H_2_O,nZVINs)_]

#### Crystal structure

Powder XRD analysis was performed to evaluate the crystallinity, phase purity, composition and crystallite size of His@nZVINs. The XRD patterns (Fig. 5b) confirmed the crystalline nature of the material, with prominent diffraction peaks at 44.69° and 65.19° corresponding to the (110) and (200) lattice planes of crystallized Fe^0^,^63^ indicating a body-centered cubic (bcc) structure.^64^ Minor diffraction peaks in the 2*θ* range of 36–47° suggested the presence of iron oxide phases,^64^ particularly Fe_3_O_4_ (36.36°, (311))^65^ and γ-Fe_2_O_3_ (38.41° and 46.81°),^66^ indicating partial surface oxidation of His@nZVINs.

The primary diffraction peak of Ni^0^ at 2*θ* = 44.5° (111) was obscured by overlap with the dominant Fe^0^ peak, while the weaker Ni^0^ reflections at approximately 52° (200) and 76.5° (220) were almost invisible due to its low content.^47^ The appearance of a diffraction peak at 2*θ* = 26.94° (114) corresponding to crystalline l-His^67^ further confirmed successful surface functionalization. The relatively noisy diffractogram can be ascribed to the magnetic nature of His@nZVINs, which reduced the signal-to-noise ratio.^48^ The average crystallite size, estimated using the Scherrer equation,^40^ was 16 nm, indicating the nanoscale nature of the material. These findings support the formation of a nanocrystalline l-His-functionalized Fe^0^ core–thin Ni^0^ shell structure.

#### Magnetic properties

VSM analysis (Fig. 5c) revealed that His@nZVINs exhibit soft ferromagnetic behavior,^48^ as evidenced by an S-shaped hysteresis loop with a saturation magnetization (*M*_s_) of 51.32 emu g^−1^, remanent magnetization (*M*_r_) of 7.93 emu g^−1^, and coercivity (*H*_c_) of 224.33 Oe. The *M*_s_ value of His@nZVINs was higher than that of chitosan-supported nZVINs,^46^ comparable to that of Fe_*x*_Ni_(1−*x*)_ alloys,^68^ and lower than that of bare nZVINs,^48^ reflecting differences in size, composition, morphology, and structural disorder. The relatively high *H*_c_ in His@nZVINs, despite their small crystallite size, can be attributed to strong intra- and interparticle dipolar interactions and structural defects.^65^ The retention of magnetic properties after functionalization indicates preservation of the metallic core, enabling efficient magnetic separation of His@nZVINs from the dispersion using a hand-held magnet.

#### Surface charge

The zeta potential (*ζ*) as a function of pH (Fig. 5d) indicated that the point of zero charge (pH_PZC_), corresponding to the isoelectric point of His@nZVINs, was 7.0. The pH_PZC_ of an adsorbent is a key parameter to figure out the adsorption mechanism. At solution pH values below the pH_PZC_, the surface of His@nZVINs became positively charged, as reflected by a positive *ζ*-potential, due to protonation of l-His amine groups (–NH_3_^+^). In contrast, at pH values above the pH_PZC_, deprotonation of l-His carboxyl and amine groups resulted in a negatively charged surface, as indicated by a negative *ζ*-potential. These observations confirmed the amphoteric nature of His@nZVINs and demonstrated the presence of a pH-responsive, chemically active surface.

### Adsorption performance

#### Preliminary comparative adsorption of E123 and E151 under unoptimized conditions

A preliminary adsorption study was conducted under identical, unoptimized conditions (20 µM dye, ambient pH and temperature, 0.5 g per L dose, equilibrium time) to evaluate dye removal feasibility and compare the adsorption performance of His@nZVINs toward the two model dyes. Slightly higher removal efficiency (*R*% = 99.40%), adsorption capacity (*q*_e_ = 37.0 mg g^−1^), and equilibrium adsorption time (*t*_eq_ = 60 min) were obtained for E151 than for E123 (*R*% = 97.60%, *q*_e_ = 22.4 mg g^−1^, and *t*_eq_ = 25 min).

These differences arise from structural variations between the dyes. The higher *R*(%) and *q*_e_ for E151 can be attributed to its greater charge density (tetra-anionic), higher hydrophobicity (five benzene rings), and enhanced hydrogen-bonding capability (diazo, tetrasulfonate, –OH, and –NHCO– moieties) compared with E123, which possesses fewer functionalities (tri-anionic, monoazo, trisulfonate, four aromatic rings, and –OH). These multiple charged, hydrophobic, and polar groups facilitate stronger interactions (*e.g.*, electrostatic, π, and hydrogen-bonding) with His@nZVINs, as further supported by subsequent pH, FT-IR and desorption studies. In contrast, the shorter *t*_eq_ for E123 is likely due to its smaller molecular size, which enables faster mobility toward active sites, consistent with kinetic studies. Overall, molecular size, charge, and functional group chemistry play a key role in governing adsorption behavior of His@nZVINs.

#### Influence of pH

**Fig. 6:**
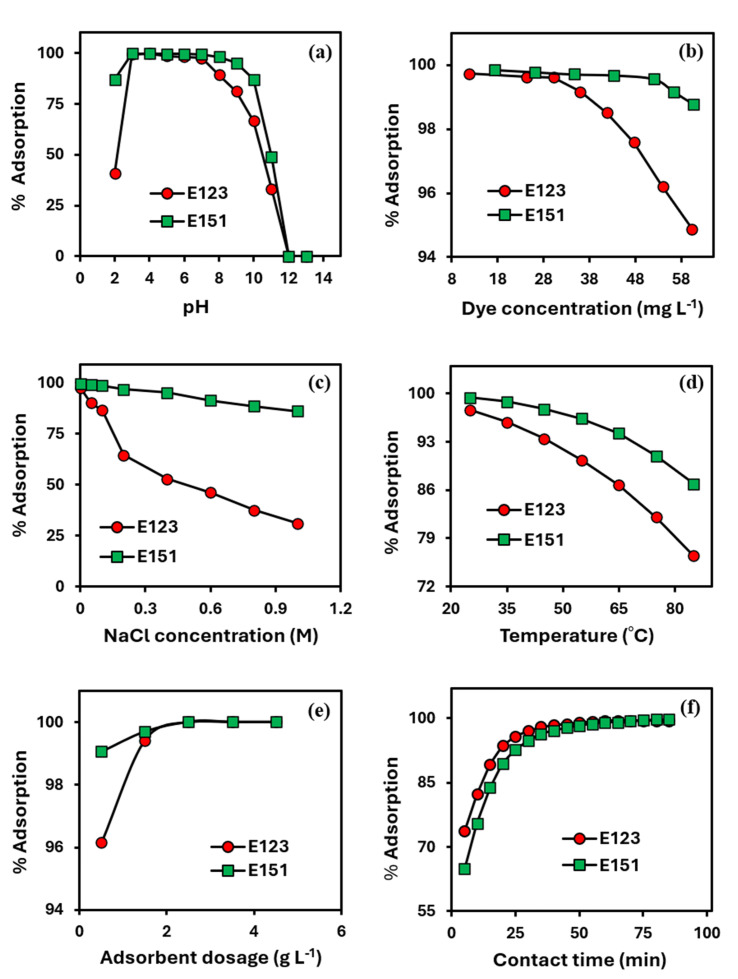
Single-factor effect on E123 and E151 removal by His@nZVINs. (a) Impact of pH. (b) Impact of initial dye concentration. (c) Impact of electrolyte. (d) Impact of temperature. (e) Impact of His@nZVINs dose. (f) Impact of exposure time.

The pH study (2–13, Fig. 6a) revealed a strong influence on dye removal. Increasing the solution pH from 4 to 13 (20 µM dye, 25 °C, 0.5 g per L dose) markedly reduced the equilibrium removal efficiency of His@nZVINs from 99.73% to 33.33% for E123 and from 99.85% to 48.96% for E151. The equilibrium adsorption capacity followed a similar trend, decreasing from 24.3 to 8.7 mg g^−1^ for E123 and from 36.1 to 15.8 mg g^−1^ for E151. No adsorption was observed above pH 11 for either dye.

A slight reduction in adsorption at pH 3 (relative to pH 4), followed by a sharp decline at lower pH, suggests adsorbent instability below pH 4, likely due to partial degradation or detachment of protonated l-His. The overall decrease in adsorption from acidic to alkaline conditions implies that this process is governed primarily by electrostatic interactions. Below the isoelectric point (pH_PZC_ = 7), protonated –NH_3_^+^ groups on the adsorbent enhance its electrostatic attraction toward anionic sulfonate (–SO_3_^−^)-containing dyes, whereas above pH_PZC_, electrostatic repulsion between the negatively charged adsorbent surface and anionic dye molecules predominates.^14,57^ Additionally, the race among excess OH^−^ ions and anionic dye molecules for adsorbent sites further inhibits adsorption in an alkaline medium.^10^

Notably, the high *R*(%) for E123 and E151 (97.6–99.4%) at the pH_PZC_ suggests that non-electrostatic factors, such as hydrophobic π–π contacts and hydrogen bonding, also contribute significantly to dye adsorption.

Overall, pH 4 was identified as optimal for both dyes because it provided the strongest electrostatic attraction with His@nZVINs. However, the substantial adsorption across pH 3–9 highlights practical applicability of His@nZVINs over the wide pH range typical of wastewater systems.

#### Influence of dye initial concentration

As the initial dye concentration increased over the tested range (E123, 11.7–60.3 mg L^−1^; E151, 17.3–60.7 mg L^−1^) while fixing all other variables (pH 4, 25 °C, 0.5 g per L dose), the equilibrium removal efficiency of His@nZVINs decreased from 99.73% to 94.88% for E123 and from 99.85% to 98.76% for E151 (Fig. 6b). In contrast, the adsorption capacity showed a substantial increase from 23.0 to 118.7 mg g^−1^ for E123 and from 38.3 to 121.0 mg g^−1^ for E151 on increasing dye concentration.

The decline in removal efficiency at higher concentrations is attributed to the limited number of available adsorption sites relative to the excess dye molecules, leading to adsorbent surface saturation and reduced *R*(%).^14,46^ Conversely, the increased *q*_e_ at elevated concentrations is driven by the higher concentration gradient between the bulk solution and the adsorbent surface, which enhances the mass transfer driving force and promotes greater dye uptake per unit mass of adsorbent.^31^

The decrease in *R*(%) exhibited a nonlinear trend, remaining relatively stable at lower concentrations (≤36 mg L^−1^ for E123 and ≤52 mg L^−1^ for E151), followed by a more pronounced decline at higher concentrations. This transition suggests that these concentrations represent a threshold beyond which active site saturation becomes significant. Accordingly, concentrations ≤36 mg L^−1^ (E123) and ≤52 mg L^−1^ (E151) may be considered optimal for near-complete removal under the studied conditions. The higher threshold and smaller decline in *R*% for E151 compared to E123 further suggest a stronger affinity of His@nZVINs for E151, likely due to differences in the molecular structure.

#### Influence of electrolyte

As the NaCl concentration increased from 0 to 1 M (20 µM dye, ambient pH, 25 °C, 0.5 g per L dose), the equilibrium adsorption efficiency and capacity of His@nZVINs decreased significantly for both dyes (Fig. 6c). The *R*(%) declined from 97.60% to 31.08% for E123 and from 99.40% to 86.13% for E151, while the *q*_e_ decreased from 22.3 to 7.7 mg g^−1^ for E123 and from 37.0 to 28.0 mg g^−1^ for E151.

This reduction is mainly attributed to the combined effects of increased ionic strength, including (i) competition between Cl^−^ ions and dye anions for available adsorption sites on the weakly cationic adsorbent surface at the applied pH (ambient pH ≈ 6.7; pH_PZC_ = 7.0) and (ii) compression of the electrical double layer and charge shielding at the solid–liquid interface by salt ions. Together, these effects reduce the effective surface potential and weaken electrostatic attraction between the adsorbent and the anionic dyes, thereby reducing dye uptake.

The more pronounced suppression observed for E123 suggests that its adsorption is more strongly dependent on electrostatic interactions, whereas E151 appears to be stabilized by a larger contribution from additional non-electrostatic interactions (*e.g.*, hydrophobic or π–π contacts), which make its uptake less sensitive to electrolyte addition. This difference can be rationalized by their charge and molecular characteristics: although E151 carries a higher negative charge (tetra-anionic) than E123 (tri-anionic), the larger molecular framework of E151 likely enables stronger multipoint interactions with the adsorbent surface, enhancing its overall binding stability. In contrast, the smaller size and lower net charge of E123 may result in weaker overall interactions and greater susceptibility to competition from Cl^−^ ions for adsorption sites, thereby facilitating its displacement at elevated ionic strength.

Therefore, low or negligible electrolyte concentration (≤0.05 M NaCl) is favorable for maintaining efficient adsorption. However, the negative impact of electrolyte at higher ionic strengths can be mitigated by operating under acidic conditions, particularly at pH 4, by enhancing the positive charge on the adsorbent surface and strengthening its electrostatic attraction toward anionic dye molecules.

#### Influence of temperature

Raising the temperature from 25 to 85 °C (20 µM dye, ambient pH, 0.5 g per L dose) reduced the dye removal performance of His@nZVINs for both dyes (Fig. 6d). The equilibrium adsorption efficiency decreased from 97.60% to 76.47% for E123 and from 99.40% to 86.87% for E151, while the corresponding adsorption capacities declined from 22.5 to 17.6 mg g^−1^ and from 38.1 to 33.3 mg g^−1^, respectively. This shows that adsorption is optimal at ambient temperature (∼25 °C).

The reduced adsorption at higher temperatures is attributed to weakened adsorbent–adsorbate interactions and enhanced dye desorption, driven by increased thermal energy and possible expansion of adsorbent active sites. These findings are consistent with an exothermic adsorption process.^37^

E151 adsorption was less prominently affected (the smaller reduction) by temperature, suggesting greater thermal stability of its adsorption compared to E123. This behavior is attributed to the larger molecular size and higher negative charge of E151, which promote its stronger binding interactions and limit mobility, thereby sustaining adsorption at elevated temperatures.^69^

#### Influence of adsorbent dosage

The effect of His@nZVINs dosage (0.5–4.5 g L^−1^) on the adsorption of E123 and E151 (25 µM dye, ambient pH, 25 °C) is shown in Fig. 6e. Increasing the adsorbent dose from 0.5 to 1.5 g L^−1^ enhanced equilibrium dye removal from 96.15% to 99.42% for E123 and from 99.06% to 99.70% for E151. A further increase in dosage resulted in only marginal improvement, and beyond 1.5 g L^−1^, the *R*(%) remained approximately constant (∼100%) for both dyes. In contrast, adsorption capacity decreased significantly as the dosage increased from 0.5 to 4.5 g L^−1^, declining from 28.9 to 3.3 mg g^−1^ for E123 and from 47.0 to 5.3 mg g^−1^ for E151.

The increase in *R*(%) with increasing adsorbent dosage is attributed to the greater availability of adsorption sites and surface area for dye molecules.^49^ The plateau observed above 1.5 g L^−1^ indicates that adsorption sites are present in excess relative to the dye concentration, resulting in negligible further improvement in removal efficiency. Conversely, the decrease in *q*_e_ with increasing dosage arises from the distribution of a fixed amount of dye over a larger adsorbent mass, resulting in incomplete utilization of available adsorption sites and thus reduced solute loading per unit mass at higher dosages.

Based on these findings, 1.5 g L^−1^ was identified as the optimal adsorbent dosage, as it enables near-complete removal of both dyes under the studied conditions without unnecessary excess adsorbent. However, 0.5 g L^−1^ was employed in most experiments to minimize adsorbent usage while still maintaining high *R*(%) and relatively higher *q*_e_.

#### Contact time effect

The impact of contact time on the adsorption of model dyes by His@nZVINs was examined for all runs under the single-factor studies (dye concentration, pH, salt concentration, temperature, and adsorbent dosage). Representative data (*R*% *versus* time) from the dosage study at 1.5 g L^−1^ (25 µM dye, 25 °C, ambient pH) for E123 and E151 are shown in Fig. 6f. Adsorptive removal of both dyes was rapid during the first 10 min, achieving 82.30% for E123 and 75.47% for E151. Removal efficiency reached ≥90% within 20 min, after which it increased gradually until equilibrium was attained, beyond which no significant further adsorption was observed. Equilibrium was reached at approximately 55 min for E123 (*R*% = 99.42%) and 70 min for E151 (*R*% = 99.70%).

The rapid initial removal is attributed to the abundance of available active sites on His@nZVINs with a high concentration gradient between the bulk dye solution and the adsorbent surface,^54^ reflecting the dominance of film diffusion at the early stage.^14^ The subsequent slower increase in *R*(%) indicates progressive saturation of the adsorbent surface and greater diffusional resistance, with adsorption progressively governed by surface rearrangement and/or intraparticle diffusion near equilibrium. No adsorption after equilibrium occurred due to occupation of all accessible adsorption sites, preventing further dye entry into the adsorbent molecular structure.

Importantly, the adsorption equilibrium time (*t*_eq_) was markedly varied with the operating conditions. For E123, *t*_eq_ increased from 15 to 120 min with increasing dye concentration (11.7–60.3 mg L^−1^), from 15 to 90 min with increasing pH (2–13), and from 25 to 120 min with increasing salt concentration (0–1 M). In contrast, *t*_eq_ decreased from 25 to 15 min with increasing temperature (25–85 °C) and from 60 to 12 min with increasing adsorbent dosage (0.5–4.5 g L^−1^). A similar pattern was observed for E151: *t*_eq_ increased from 20 to 150 min with dye concentration (17.3–60.7 mg L^−1^), from 20 to 120 min with pH (2–13), and from 60 to 150 min with salt concentration (0–1 M), whereas it decreased from 60 to 35 min with increasing temperature (25–85 °C) and from 80 to 28 min with increasing adsorbent dosage (0.5–4.5 g L^−1^). Collectively, the *t*_eq_ spanned 12–120 min for E123 and 20–150 min for E151.

These results indicate that the shortest *t*_eq_ for His@nZVINs was achieved at higher temperature and adsorbent dosage, while higher pH, salt concentration, and dye concentration prolonged equilibration. The observed variation in *t*_eq_ arises from multiple interacting factors. Higher dye concentrations increase the dye-to-site ratio on His@nZVINs, causing steric hindrance, ion competition, and repulsion between adsorbed and free dye molecules, which retard diffusion and prolong *t*_eq_. Similarly, alkaline pH and higher ionic strength promote competitive adsorption of OH^−^ or Cl^−^ ions in anionic dye systems and weaken electrostatic dye–His@nZVINs interactions, resulting in slower adsorption and longer *t*_eq_. In contrast, elevated temperature enhances molecular mobility, and higher adsorbent dosage increases the availability of accessible active sites, both of which make it easier for dye molecules to bind to the surface, accelerating dye adsorption and shortening *t*_eq_. The consistently faster equilibration of E123 compared with E151 is related to its smaller size and lower degree of functionalization, which favor faster mass transfer. Overall, the adsorption kinetics are governed by the combined effects of these factors rather than by a single controlling factor. Rapid equilibration and negligible post-equilibrium desorption across all operating conditions further showcase the robustness and practical suitability of His@nZVINs for food azo dye removal on larger scales.

#### E123 and E151 removal in real water matrices: practical applicability of His@nZVINs

To evaluate (i) the practical applicability of His@nZVINs under environmentally relevant conditions and (ii) the matrix effects arising from varying levels of naturally occurring dissolved salts and competing ions on dye removal, adsorption experiments were conducted in four real water matrices (drinking water, tap water, groundwater, and seawater), using deionized (DI) water as the reference. Experiments were performed at initial dye concentrations of 20 and 50 µM (ambient pH and temperature, 0.5 g per L dose, equilibrium time). To characterize matrix complexity in real waters, key water quality parameters, including total dissolved solids (TDS), salinity, electrical conductivity (EC), and major inorganic ions (Cl^−^, SO_4_^2−^, HCO_3_^−^, NO_3_^−^, Na^+^, Ca^2+^, Mg^2+^, and K^+^), were also measured (Table S2).

**Fig. 7:**
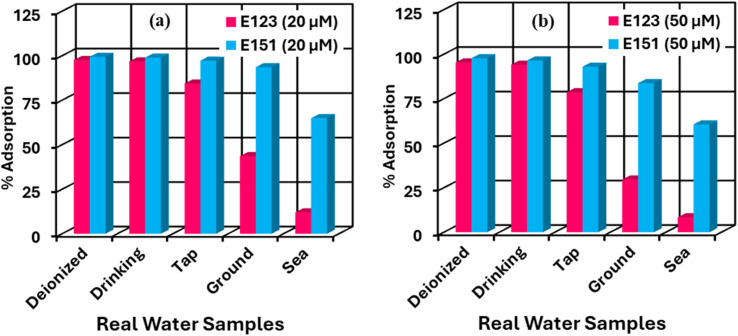
Impact of real water matrices on elimination of E123 and E151 by His@nZVINs at an initial dye concentration of (a) 20 µM and (b) 50 µM.

As shown in Fig. 7, E123 removal efficiencies at 20 and 50 µM were 97.60% and 95.37% in DI water, 96.81% and 94.10% in drinking water, 84.41% and 78.65% in tap water, 43.62% and 29.61% in groundwater, and 12.03% and 8.40% in seawater, respectively. For E151, the corresponding *R*(%) values at 20 and 50 µM were 99.40% and 97.79% in DI water, 98.94% and 96.50% in drinking water, 97.27% and 92.98% in tap water, 93.56% and 83.75% in groundwater, and 64.95% and 60.57% in seawater. Water quality analysis revealed a progressive increase in salinity (168–38 420 ppm), EC (322–53,009 µS cm^−1^), and TDS (160–26 500 ppm) from drinking water to seawater (Table S2). A similar increasing trend was observed for major ions, except HCO_3_^−^ and NO_3_^−^, which were higher in groundwater than in seawater.

The results demonstrate a strong matrix-dependent adsorption behavior for both dyes, where *R*(%) decreased with increasing matrix complexity or ion richness in the following order: DI water ≈ drinking water > tap water > groundwater > seawater. The marked decline in groundwater and seawater is attributed to their high ionic strength, reflected by elevated salinity, EC, and TDS, which in turn indicates the presence of abundant competing ions. In particular, high levels of Cl^−^ (≥1596 ppm), SO_4_^2−^ (≥210 ppm), and HCO_3_^−^ (≥94 ppm) in these waters likely competed with dye anions for adsorption sites and caused charge shielding, thereby weakening dye–His@nZVINs electrostatic attractions. Likewise, high concentrations of dissolved cations, such as Na^+^ (1010–8406 ppm), Ca^2+^ (120–349 ppm), and Mg^2+^ (117–797 ppm), in groundwater and seawater may have further suppressed dye adsorption through coordination with N-, S- or O-donor functional groups on dyes and/or the His@nZVINs surface, thereby blocking active sites.

The matrix interference was much more pronounced for E123 than for E151. E123 removal dropped sharply in groundwater and became very low in seawater, whereas E151 retained high removal in groundwater and moderate removal even in seawater (Fig. 7). This suggests the stronger affinity of E151 for His@nZVINs than E123, consistent with the results of our controlled salt study.

Overall, His@nZVINs showed high efficacy in low-ionic-strength waters and remained particularly promising for E151 removal even in complex real-water matrices. Although performance declined in highly saline or ion-rich waters, especially for E123, this matrix effect can be mitigated under optimized conditions (*e.g.*, pH 4, higher adsorbent dose, and pre-treatment/desalting), supporting the practical applicability of His@nZVINs for water treatment.

### Adsorption isotherms, kinetics, and thermodynamic modeling

#### Isotherms

**Fig. 8:**
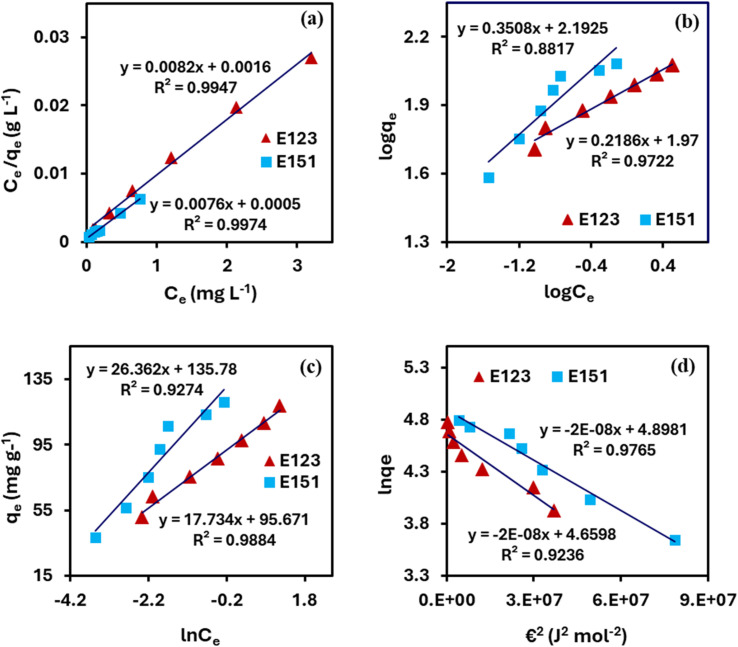
Isothermal models for E123 and E151 adsorption onto His@nZVINs: (a) Langmuir. (b) Freundlich. (c) Temkin. (d) Dubinin–Radushkevich.

Adsorption isotherms provide quantitative evaluation of adsorption with a comprehensive understanding of adsorbate distribution from liquid to the solid adsorbent surface at equilibrium.^59^ Determining the most suitable correlation from isothermal models is important in constructing an improved adsorption setup for dye removal and elucidating the predominant type of adsorbent–adsorbate interaction. Four widely recognized isothermal models (Langmuir, Freundlich, Dubinin–Radushkevich, and Temkin), applied in this research, are represented with their mathematical linear equations and plotting parameters in Table S1. Important statistical parameters from the slopes and intercepts of isothermal plots of E123 and E151 (Fig. 8), drawn using equilibrium adsorption data of varying initial dye concentrations (11.7–60.7 mg L^−1^) under optimal conditions (pH = 4, temperature = 25 °C, adsorbent dosage = 0.5 g L^−1^), are given in Table 1.

**Table 1:** Isothermal variables for the adsorption of E123 and E151 onto His@nZVINs

Isothermal models	Parameters	E123	E151
Langmuir	*K*_L_ (L mg^−1^)	5.125	15.20
	*R*_L_	0.006–0.014	0.001–0.004
	*q*_max_ (mg g^−1^)	122.0	131.6
	*R* ^2^	0.995	0.997
Freundlich	*n*	4.575	2.851
	*K*_F_ (mg^1−1/*n*^ L^1/*n*^ g^−1^)	93.32	155.8
	*R* ^2^	0.972	0.882
Dubinin–Radushkevich	*K*_DR_ (mol^2^ J^−2^)	2 × 10^−8^	2 × 10^−8^
	*q*_m_ (mg g^−1^)	105.6	134.0
	*E* (kJ mol^−1^)	5.000	5.000
	*R* ^2^	0.924	0.977
Temkin	*A* (L g^−1^)	220.2	172.5
	*b* (J mol^−1^)	139.7	93.98
	*R* ^2^	0.988	0.927

Isothermal study indicated that both model dyes adsorb onto His@nZVINs in full compliance with the Langmuir isotherm as its *R*^2^ value (0.995–0.997) was highest and approaching unity (Fig. 8a), in contrast to all other models (*R*^2^ < 0.99). The Langmuir model implies that the dye molecules adsorb in a monolayer onto the homogeneous adsorbent surface of energetically similar binding sites, with no lateral interactions among the absorbed molecules and binding of one adsorbate molecule per site on adsorbent.^16,31^ The estimated maximum monolayer adsorptive capacities (*q*_max_) for E123 (122.0 mg g^−1^) and E151 (131.6 mg g^−1^) closely match the respective experimental adsorption capacity values (*q*_exp_, 118.6 mg g^−1^ for E123 and 121.03 mg g^−1^ for E151). The slightly lower adsorption capacity for E123 compared to E151 demonstrates the stronger tendency of E151 to bind to the His@nZVINs surface. This difference for the two dyes is ascribed to variations in their structure-related physicochemical properties, including composition, functional groups, and charge, which govern dye–adsorbent interactions and thus drive the adsorption process. The values of *K*_L_ (adsorption free-energy-related Langmuir constant) for E123 (5.125 L mg^−1^) and E151 (15.20 L mg^−1^) indicate that E151 has a stronger binding strength or affinity than E123.^46^ The dimensionless Langmuir separation factor [*R*_L_ = 1/(1 + *K*_L_*C*_0_)] ranged at 0.006–0.014 for E123 and 0.001–0.004 for E151 across the applied concentration range. Since 0 < *R*_L_ < 1, adsorption of both anionic dyes is favorable.^16^

The Freundlich model postulates arrangement of adsorbate molecules as multiple layers on a heterogeneous adsorbent surface of variable affinity and energy with mutual interactions of adsorbate molecules, with powerful sites being populated first with lowering of binding affinity on increased site occupation.^37^ The important Freundlich constant *K*_F_ (adsorptive capacity in mg^1−1/*n*^ L^1/*n*^ g^−1^) was calculated to be 93.32 for E123 and 155.8 for E151. The dimensionless Freundlich constant *n* (heterogeneity factor indicating adsorption strength) obtained from the Freundlich isotherm (Fig. 8b and Table 1) lies within 2–10, indicating strong dye–His@nZVINs interactions and favorable adsorption, particularly at high dye concentrations.^10^ The Freundlich model yielded *R*^2^ values of 0.972 (E123) and 0.882 (E151), indicating its poorer fit of adsorption data than the Langmuir model (*R*^2^ > 0.99) for both dyes, but it fits E123 adsorption better than E151.

The Temkin model assumes temperature-dependent linear drop in adsorption heat experienced by each adsorbate molecule in layer on increasing coverage, with uniform binding energy distribution up to a certain optimal level, being solely true for moderate adsorbate concentrations.^10^ The computed constants *A* and *b* (Table 1) from the Temkin model (Fig. 8c) correspond to maximal binding energy and heat of adsorption, respectively. The Temkin model (*R*^2^ of 0.988 for E123 and 0.927 for E151) outperforms the Freundlich model (*R*^2^ = 0.882–0.972) but fits the adsorption data less agreeably than the Langmuir model (*R*^2^ > 0.99) for both dyes. The values of *b* (93.98–139.7 J mol^−1^) are low, suggesting physisorptive interactions among dyes and adsorbent active sites.

The Dubinin–Radushkevich (D–R) isothermal model explains the adsorption mechanism on both heterogeneous and homogeneous surfaces and is used to estimate adsorption free energy and characteristics. The parameters *K*_DR_ and *q*_m_ (Table 1) correspond to the D–R isothermal constant and predicted monolayer saturation adsorption capacity. The Polanyi potential (*€*^2^) for the D–R isothermal plot (Fig. 8d) and related average free energy of adsorption (*E*) are calculated using eqn (6) and (7), respectively.^59^

6 *€* = *RT* ln(1 + 1/*C*_e_)

7 *E* = 1/√2*K*_DR_

The value of *E* provides insight into the adsorption mechanism: physisorption (*E* < 8 kJ mol^−1^), ion-exchange (*E* = 8–16 kJ mol^−1^), and chemisorption (*E* > 16 kJ mol^−1^).^59^ The calculated *E* value for both E123 and E151 is 5 kJ mol^−1^, suggesting that their adsorption is predominantly governed by physical interactions. The D–R model shows the lowest *R*^2^ value (0.924) for E123 among all models, indicating a poor fit to its experimental adsorption data. In contrast, for E151, the D–R model provides a relatively high *R*^2^ value (0.977), ranking second after the Langmuir model and exceeding those of the Temkin and Freundlich models.

Overall, the Langmuir model provided the best fit, indicating uniform monolayer adsorption of E123 and E151 on the homogeneous surface of His@nZVINs, attributed to the homogeneous distribution of l-His *via in situ* functionalization, as confirmed by EDX elemental mapping. As adsorption of these model dyes using nZVINs has been rarely reported, Table 2 presents a comparison of *q*_max_ of His@nZVINs with many other reported adsorbents for E123 and E151, along with some amino acid-modified and nZVIN-based systems for other azo dyes under various experimental conditions. The compiled data show that His@nZVINs exhibit relatively higher adsorption capacities than many reported adsorbents. However, such comparisons should be interpreted with caution, as variations in key experimental parameters (*e.g.*, pH, temperature, adsorbent dose, and dye concentration) can significantly affect *q*_max_ values. Thus, the apparent superiority of His@nZVINs cannot be generalized across all systems; however, more meaningful comparisons can be made with studies conducted under relatively similar conditions, for example, in the case of surfactant-modified pink clay for E123,^14^ polyelectrolyte–chitosan–magnetite for E151,^16^ chitosan–Fe/Ni systems^46,51^ for direct red 16 and direct blue 86, and AA-ionic liquid/Fe_3_O_4_–SiO_2_ (ref. 33) for Congo red. Within this context, the comparable or improved performance of His@nZVINs highlights the synergistic effect of combining Fe/Ni bimetals and l-His, demonstrating their applicative potential for efficient removal of food azo dyes from aqueous environments and encouraging further research.

**Table 2:** Comparisons of adsorptive capacity of His@nZVINs with that of previously reported adsorbents

Dye	Adsorbent	*q*_max_ (mg g^−1^)	pH	*T* (°C)	Dosage (g L^−1^)	*t*_eq_ (min)	Ref.
E123	Fe_3_O_4_–MgO NPs	38.0	9.0	30	20.0	60	55
	CTAB-pink clay	73.0	2.0	55	0.5	60	14
	ZnO NPs	75.9	6.0	35	3.0	30	10
	Aminated avocado seed	89.2	2.0	30	1.7	120	57
	Water hyacinth leaves	70.6	2.0	18	1.0	4320	70
	His@nZVINs	122.0	4.0	25	0.5	120	This study
E151	Chitosan–lignin–TiO_2_ nanocomposites	15.8	6.0	25	13.3	60	54
	Polyethyleneimine–chitosan–Fe_3_O_4_	69.0	7.0	25	1.1	30	16
	Fe-hydrochar (orange peel)	10.5	7.0	45	2.3	26	8
	Activated kaolinite	1.1	7.0	20	5.0	1440	53
	Activated pine wood	2.5	2.0	25	15.0	1440	71
	His@nZVINs	131.6	4.0	25	0.5	150	This study
Direct red 16	Chitosan–Fe/Ni	84.7	4.0	25	0.2	30	46
Direct blue 86	Chitosan–Fe/Ni	61.7	4.0	25	0.2	25	51
Methyl orange	Phenylalanine-LDH–Fe_3_O_4_/PVA	19.7	6.0	25	5.0	420	35
Congo red	Amino acid–ionic liquid/Fe_3_O_4_–SiO_2_	90.0	3.0	25	0.3	15	33
Acid red G	Alanine–TiO_2_	59.1	3.0	25	2.0	120	72

#### Kinetics

**Fig. 9:**
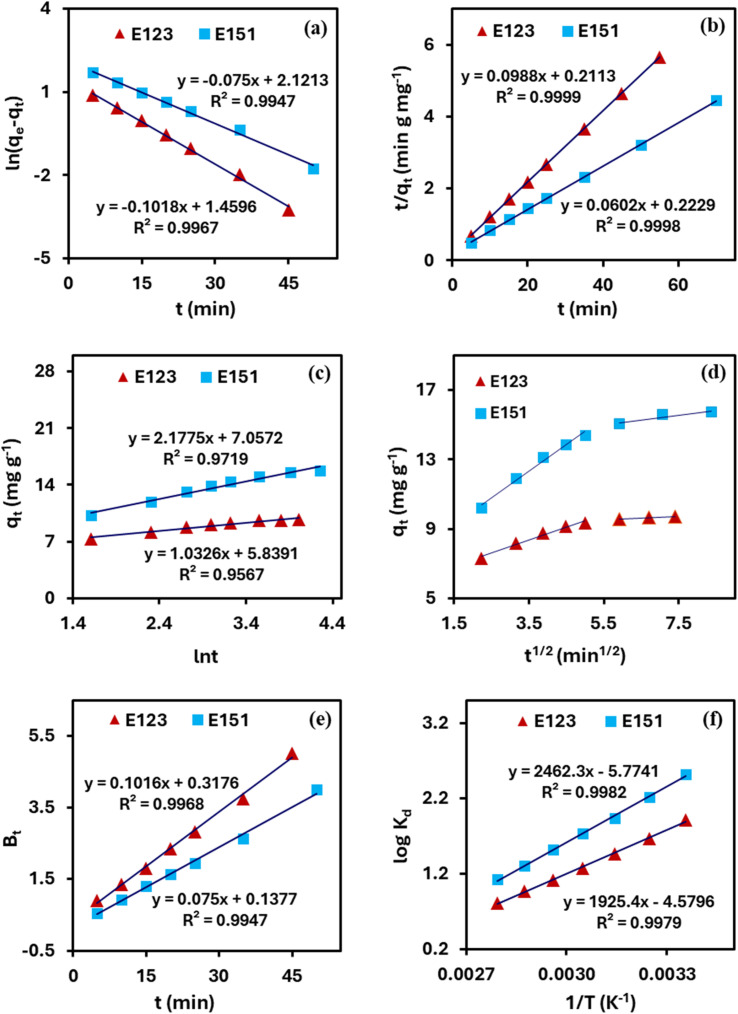
Kinetics and thermodynamics of E123 and E151 adsorption onto His@nZVINs. (a) Pseudo-1st order kinetics plots. (b) Pseudo-2nd order kinetics plots. (c) Elovich kinetics plots. (d) IPD kinetics plots. (e) Boyd kinetics plots. (f) Thermodynamics van't Hoff regression plots.

Adsorption kinetics models assist in determining pollutant removal rates and identifying the rate-limiting stages, for example, diffusion, mass transfer, and chemical reactions. For kinetic modeling, the adsorption capacity (*q*) of His@nZVINs for E123 and E151 was measured over time until equilibrium under fixed conditions (initial dye concentration of 25 µM, adsorbent dose of 1.5 g L^−1^, temperature of 25 °C, and ambient pH). The plots from five applied kinetics models (Lagergren's pseudo-1st order, pseudo-2nd order, Elovich, intraparticle diffusion, and Boyd) are displayed in Fig. 9a–e. These plots were constructed using respective linearized mathematical expressions (eqn (S1)–(S5)),^16,29^ and kinetics parameters (Table 3) were computed from plots' slopes and intercepts.

**Table 3:** Kinetics parameters for E123 and E151 adsorption on His@nZVINs

Kinetics models		Parameters	E123	E151
Experimental		*q*_e_ (mg g^−1^)	9.71	23.53
Pseudo-1st order		*q*_e_ (mg g^−1^)	4.30	8.34
		*k*_1_ (min^−1^)	0.102	0.075
		*R* ^2^	0.9967	0.9947
Pseudo-2nd order		*q*_e_ (mg g^−1^)	10.12	16.61
		*k*_2_ (g mg^−1^ min^−1^)	0.046	0.016
		*R* ^2^	0.9999	0.9998
Elovich		*a* (g mg^−1^ min^−1^)	295.0	54.77
		*b* (g mg^−1^)	0.968	0.460
		*R* ^2^	0.9567	0.9719
Intraparticle diffusion	I	*C*_i_ (mg g^−1^)	5.726	7.030
		*k*_id_ (mg g^−1^ min^−1/2^)	0.753	1.515
		*R* ^2^	0.9823	0.9867
	II	*C*_i_ (mg g^−1^)	9.033	13.45
		*k*_id_ (mg g^−1^ min^−1/2^)	0.093	0.282
		*R* ^2^	0.9547	0.9038
Boyd		*R* ^2^	0.9968	0.9947

According to the pseudo-1st order kinetics model, the difference between the solute mass adsorbed over time and concentration at equilibrium is in direct proportion to the rate at which solute adsorption changes over time, generally applicable during the initial adsorption phase. The pseudo-2nd order kinetics model, on the other hand, presumes that the solute uptake rate follows second order in relation to the available surface sites, considering the overall rate effect, and it suggests chemisorption as the rate-controlling mechanism.^10^ The higher *R*^2^ values (>0.999) and better accord among estimated and empirical *q*_e_ values for the pseudo-2nd order model (Table 3 and Fig. 9b), compared with the pseudo-1st order model (*R*^2^ = 0.995–0.997; Fig. 9a), evidence a good conformity of adsorption of model dyes on His@nZVINs to pseudo-2nd order kinetics. Nevertheless, the consistently high *R*^2^ values (>0.99) obtained for the pseudo-1st order model also demonstrate good linearity, implying that the adsorption of E123 and E151 proceeds *via* a complex mechanism involving multiple concurrent processes rather than a single rate-controlling step. The estimated rate constants of pseudo-1st order kinetics (*k*_1_) and pseudo-2nd order kinetics (*k*_2_) were slightly higher for E123 (*k*_1_ = 0.10 min^−1^ and *k*_2_ = 0.05 g mg^−1^ min^−1^) than for E151 (*k*_1_ = 0.08 min^−1^ for *k*_2_ = 0.02 g mg^−1^ min^−1^), indicating faster adsorption of E123. This behavior is attributed to smaller molecular size and fewer functional groups of E123 for interaction, which facilitate more rapid access to available active adsorbent sites with fast equilibrium. As the *k*_1_ values are higher than *k*_2_ for both dyes, adsorption of E123 and E151 on His@nZVINs likely follows a two-stage mechanism. Initially, a rapid uptake occurs through charge-based interactions (*e.g.*, electrostatic attraction), consistent with pseudo-first-order kinetics, followed by a slower stage governed by additional physical/non-covalent interactions (*e.g.*, π–π interactions, hydrogen bonding, and weak surface complexation), better described by pseudo-second-order kinetics. These latter interactions promote dye immobilization (stabilization) on the His@nZVINs surface, facilitating the accommodation of larger dye molecules.

The Elovich model considers activated chemisorption processes, implying that the solid surfaces possess energetic heterogeneity and that the kinetics of adsorption is not much influenced by desorption or interactions among adsorbed species, particularly when there is little surface coverage.^29,73^ The initial adsorption rate (*a*) and desorption constant (*b*) related to the surface coverage amount for model dyes (Table 3) were computed respectively from the intercept and slope of Elovich plots (Fig. 9c). Low regression coefficients (*R*^2^ < 0.98) of Elovich plots for both dyes indicate a poor match of adsorption data for this model, in comparison with pseudo-1st or pseudo-2nd order paradigms.

The theory put forward by Weber and Morris forms the origin of the intraparticle diffusion (IPD) model that explains the diffusion mechanism by considering the proportional variation of dye loading with *t*^1/2^ instead of contact time (*t*).^14^ IPD is typically the rate-governing stage of the adsorption process when dye molecules are transported from the fluid bulk into hardly accessible inner pores of the adsorbent. Fig. 9d illustrates two separate linear regions in the IPD plots; however, none of the lines cross the origin for any dye as their interceptive values (boundary layer thickness, *C*_i_) are greater than zero (5.7–13.5 mg g^−1^, Table 3). This suggests that IPD is not a single rate-controlling mechanism for model dye adsorption on His@nZVINs; instead, the adsorption process consists of two primary diffusion steps: the first linear region (I) is ascribed to the external mass transfer effect, also known as boundary or film layer diffusion, involving dye diffusion to the exterior His@nZVINs surface from the bulk and covering easily accessible surface binding sites, and the second linear segment (II) is assigned to the effect of IPD.^55^ For both dyes, the intraparticle diffusion rate constant (*k*_id_) in region I is 5–8 times higher than that in region II, whereas the dye concentration (*C*_i_) is approximately two-fold greater in region II. These differences indicate that dye transport from the bulk solution to the external surface of His@nZVINs is significantly easier (faster) than diffusion into internal sites. This behavior reflects the relatively smooth surface or nonporous nature of His@nZVINs, allowing binding of dyes only to the adsorbent surface (consistent with the Langmuir model). The larger *C*_i_ values for both regions in the case of E151 (7.03–13.45 mg g^−1^) compared to E123 (5.72–9.03 mg g^−1^) suggest that E151 adsorption is more strongly influenced by boundary layer effects, most likely due to its higher molar mass (867.7 g mol^−1^) and more functional groups, facilitating greater dye interaction and surface retention. In contrast, the smaller molar mass of E123 (604.5 g mol^−1^) with fewer functionalities induces a lower mass transfer effect and retention on the adsorbent surface. Notably, the elementary diffusion steps in both regions are 2–3 fold faster (higher *k*_id_) for E151 than for E123, despite the larger molecular size of E151. This indicates that dye diffusion to the His@nZVINs surface is not governed solely by molecular size but is driven by other structural factors, such as negative charge density and the number of functional groups, which facilitate rapid migration of dye molecules toward the adsorbent surface (particularly at shorter diffusion distances) and influence adsorption efficiency.

The Boyd kinetics model also predicts the rate-limiting stage and identifies the diffusion mechanism of adsorption. The adsorption rate is said to be controlled by pore diffusion, or IPD, if the *B*_*t*_*versus t* plot is linear with a zero intercept; *B*_*t*_ equals [−0.4977 − ln(1 − *F*)], wherein *F* = *q*_*t*_/*q*_e_. In contrast, the nonlinear plot or the plot that is linear without intersecting the origin indicates that film diffusion or external mass transport is the dominant factor influencing the adsorption process.^16,59^ Boyd model plots of E123 and E151 (Fig. 9e) with *R*^2^ values of 0.9947–0.9968 evidence their good linearity, but they do not pass through the origin. This suggests that the adsorption of both dyes on His@nZVINs follows film diffusion as a dominant mechanism.

#### Thermodynamics

To better elucidate the effect of temperature (*T*) on His@nZVINs uptake of model dyes, the thermodynamic variables (Δ*S*°, Δ*H*°, and Δ*G*°), listed in Table 4, were estimated by applying van't Hoff regression plots (Fig. 9f), drawn based on eqn (8).^46^ The Δ*G*° at various *T* is calculated using eqn (9) or (10).^37^

8 log *K*_d_ = (Δ*S*°/2.303*R*) − (Δ*H*°/2.303*RT*)

9 Δ*G*° = −*RT* ln *K*_d_

10 Δ*G*° = Δ*H*° − *T*Δ*S*°

**Table 4:** Thermodynamics data for E123 and E151 adsorption onto His@nZVINs at varying temperatures

Dye	*T* (K)	Δ*G*° (kJ mol^−1^) from eqn (9)	Δ*G*° (kJ mol^−1^) from eqn (10)	*K*_d_ (L g^−1^)	Δ*S*° (J mol^−1^ K^−1^)	Δ*H*° (kJ mol^−1^)	*R* ^2^
E123	298	−10.73	−10.90	81.33	−87.69	−36.87	0.9979
	308	−9.857	−9.796	45.85			
	318	−8.980	−8.856	28.49			
	328	−8.104	−7.993	18.75			
	338	−7.227	−7.211	13.02			
	348	−6.350	−6.402	9.142			
	358	−5.473	−5.571	6.500			
E151	298	−14.20	−14.38	331.3	−110.6	−47.15	0.9982
	308	−13.10	−13.05	163.3			
	318	−11.99	−11.77	85.72			
	328	−10.88	−10.83	53.10			
	338	−9.778	−9.797	32.66			
	348	−8.673	−8.668	20.00			
	358	−7.567	−7.687	13.23			

*K*_d_ represents the distribution coefficient (*q*_e_/*C*_e_). *R* is the ideal gas constant (8.314 J mol^−1^ K^−1^). The negative Δ*G*° values at all applied temperatures (Table 4) indicate spontaneous and thermodynamically favorable adsorption for both dyes.^14^ The thermodynamic feasibility or spontaneity declined with rising temperature, as evidenced by greater Δ*G*° values with rising temperature. The negative Δ*H*° values for both dyes describe their adsorption as exothermic,^46^ being consistent with the decreasing trend of *R*(%) or *q*_e_ on rising temperature due to weakened intermolecular attractive forces. The solid–solution interface exhibited reduced randomness attributable to slight structural modifications in the adsorbate and adsorbent during the process of model dye adsorption, as suggested by their negative Δ*S*° values.^73^ A small difference in the magnitude of thermodynamic parameters of the two dyes suggests that their adsorption on His@nZVINs occurs *via* a similar mechanism, involving the same kind of interaction but to a somewhat different extent, consistent with the experimental removal efficiencies. The values of Δ*H*° and Δ*G*° for adsorption may serve as indicators to differentiate between physisorption and chemisorption. Typically, Δ*H*° of physisorption exists below 40 kJ mol^−1^, whereas the range for chemisorption is 80–200 kJ mol^−1^. Δ*G*° typically lies from −20 to 0 kJ mol^−1^ for physisorption while from −80 to −400 kJ mol^−1^ for chemisorption.^37^ In this study, the Δ*H*° and Δ*G*° values almost fell within the physisorption range, indicating that adsorption of E123 and E151 onto His@nZVINs is predominantly driven by physical interactions. This interpretation is further supported by exothermic (inverse temperature-dependent) behavior, easy reversibility (desorption), fast diffusion-controlled kinetics, the absence of any spectral (FT-IR/electronic) evidence of new chemical bond formation, and low Temkin/D–R energies. The apparent fit to pseudo-2nd order kinetics therefore does not necessarily imply chemisorption, but rather reflects slower secondary interactions (*e.g.*, hydrogen bonding, π–π interactions, and weak surface coordination) following rapid electrostatic uptake. Overall, physisorption is identified as the dominant mechanism, consistent with previous dye adsorption studies that report low exothermic enthalpies and comparable fits to both kinetic and isotherm models.^35,37,73,74^

### Spectral insights into the adsorption mechanism

#### Electronic spectra

**Fig. 10:**
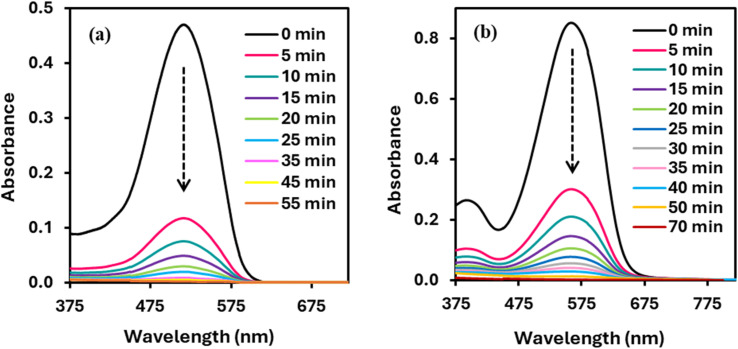
Comparative electronic spectra for the removal of (a) E123 and (b) E151 by His@nZVINs at varying time intervals under fixed empirical conditions: initial dye concentration = 25 µM, adsorbent mass = 1.5 g L^−1^, pH = ambient, temp. = 298 K.

The electronic spectra of model dyes before and after adding His@nZVINs at different time intervals were compared to learn more about the underlying mechanism of dye removal. Fig. 10a and b show the time-dependent visible spectra for the removal of E123 and E151, respectively, under the fixed conditions stated in the figure description. The strongest absorption band in the visible spectra characterizes the dye chromophore (–N=N–). Both dyes exhibit a reduction in the band intensity (to a certain extent) over time after His@nZVINs addition until equilibrium is achieved, affirming their removal from water. Unlike previous dye removal studies with nZVINs,^49,50^ the electronic spectral shapes for both dyes remained unchanged (except for a drop in band intensity) after exposure to His@nZVINs; neither the dye *λ*_max_ was altered nor the development of new bands for newly created species in solution was evidenced here. In the absence of any induced redox agent (oxidant/reductant) or light exposure, these electronic spectral outcomes confirm that removal of both model dyes by His@nZVINs occurs solely *via* the adsorption phenomenon, ruling out the chance of sole or concurrent dye degradation (photo- or catalytic), which typically causes substantial spectral alteration.^49,50,75,76^ This conclusion is further supported by controlled UV irradiation experiments, which showed neither spectral changes nor enhanced removal efficiency, and by desorption studies, where recovery of the original dye color and characteristic absorption bands in the desorbing media reaffirmed the adsorption mechanism. The dominant dye adsorption rather than catalytic reduction (degradation) by His@nZVINs likely occurs owing to the synergistically designed interface. The deposition of Ni over the Fe core, together with *in situ*l-His functionalization, may partially block Fe/Ni electron transfer sites while firmly anchoring small l-His onto the core–shell structure. This creates a tailored surface enriched with multiple functional groups (as verified by FT-IR studies), favoring molecular recognition and hence stronger dye adsorption (consistent with higher *q*_max_) while suppressing the intrinsic catalytic redox activity of nZVINs (mostly reported for bare or chitosan-functionalized nZVINs).^46,49^ Moreover, there was no proof of the formation of any aggregates of dyes with His@nZVINs, linked to significant intermolecular pi–pi or coulombic (charge) interactions that could normally be identified through a discernible blue or red shift in the absorption spectrum of the dye.^73^

#### FT-IR spectra

**Fig. 11:**
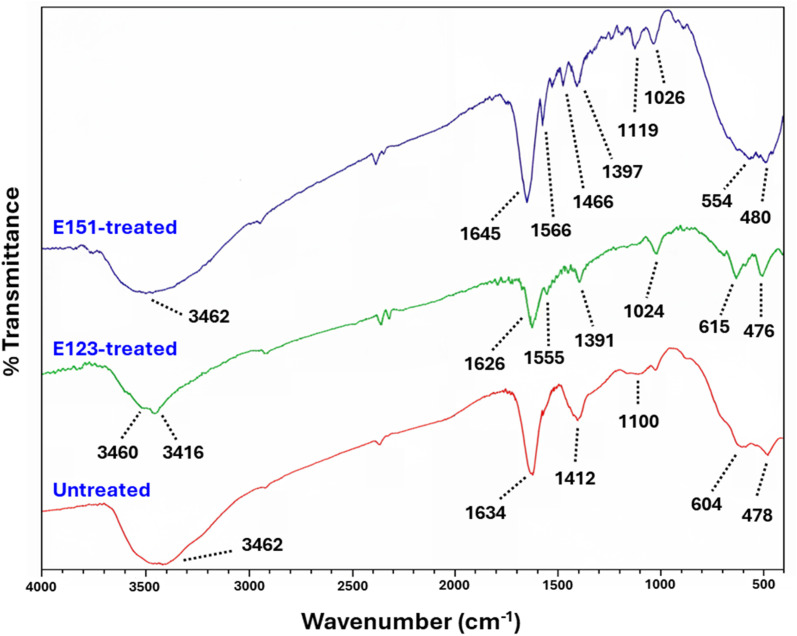
FT-IR spectral comparison of untreated, E123-treated, and E151-treated His@nZVINs.

For precise mechanistic insights into the dye–His@nZVINs interactions, FT-IR spectra of His@nZVINs before and after treatment with E123 and E151 were compared with each other (Fig. 11) and with the spectra of free dyes (SI Fig. S3 and 4). Relative to pristine His@nZVINs, E123 treatment led to an overall decrease in band intensities, whereas E151 treatment caused pronounced broadening in certain bands, with noticeable shifts in many bands for both cases, confirming dye adsorption involving multiple interactions. The presence of some new dye-related bands for E151 (less evident for E123, likely due to lower surface coverage or stronger binding) further supports dye adsorption.

In detail, the carboxylate *ν*_s_/*ν*_as_ bands (1412/1634 cm^−1^) of His@nZVINs shifted by 8–21 cm^−1^ (after E123 adsorption) and ±15 cm^−1^ (after E151 adsorption), suggesting involvement of –COO^−^ oxygen in hydrogen bonding interaction with dye –OH/–NH functionalities. Electrostatic attraction between adsorbent protonated –NH_3_^+^ and dye sulfonate (–SO_3_^−^) groups (consistent with pH studies) was evidenced by changes in the N–H stretching band (3462 cm^−1^), including band suppression with splitting (two peaks at 3460 and 3416 cm^−1^ after E123 adsorption) and significant band broadening (after E151 adsorption), along with disappearance of the strong –SO_3_^−^ (S=O) *ν*_as_ band (1193–1200 cm^−1^).^37^ For E151 adsorption, a new shifted band of –SO_3_^−^*ν*_s_ at 1119 cm^−1^ (relative to 1123 cm^−1^ in free E151) indicates involvement of sulfonate in further binding through hydrogen bonding (with adsorbent –NH) and/or surface coordination with Fe/Ni sites. A marked attenuation (almost disappearance) of the adsorbent imidazole C–N band (1100 cm^−1^) suggests its participation in π–π and/or n–π interactions with dye aromatic rings. These pi-interactions were further validated for E151 by the appearance of a shifted benzenoid C=C band at 1466 cm^−1^ (compared to 1499 cm^−1^ for the free dye).^72^ The Fe–O vibration (604 cm^−1^) was shifted by +11 cm^−1^ (for E123) and −50 cm^−1^ (for E151), implying coordination between Fe sites and dye oxygen-containing groups (–OH and –SO_3_^−^). The phenolic –OH involvement (complexation and hydrogen bonding) was also corroborated by considerable shift of the C–O band of E151 (1036 → 1026 cm^−1^) and E123 (1049 → 1024 cm^−1^).^37^ Additionally, the emergence of new bands at 1566 cm^−1^ (E151-treatment) and 1555 cm^−1^ (E123-treatment), assigned to azo (–N=N–) stretching, confirms that the dyes remain structurally intact during removal (consistent with electronic spectra),^73^ while their red shifts (12 and 6 cm^−1^ relative to free E151 and E123, respectively) further advocate possible coordination of azo nitrogen with metal sites. Other bands showed negligible shifts (0–2 cm^−1^), indicating no other direct involvement in binding.

These results collectively demonstrate a synergistic adsorption mechanism involving electrostatic attraction, hydrogen bonding, π-interactions, and surface coordination. Although similar carboxylate–amine interactions are reported for other amino acid-functionalized adsorbents (*e.g.*, isoleucine-, lysine-, alanine-, and phenylalanine-modified systems),^33–35,72^His@nZVINs exhibit superior performance (higher *q*_max_) due to the imidazole functionality of l-His. This polar, aromatic moiety enhances adsorption through increased surface positive charge (=NH^+^–, p*K*_a_ = 6.0–6.5), providing increased electrostatic attraction toward anionic (acidic) dyes even at ambient pH, π–π/n–π interactions, and additional hydrogen-bonding sites. Furthermore, chelate coordination of imidazole nitrogen atoms with Fe/Ni during synthesis can partially passivate metal electron-active sites *via* π-back donation, transforming the Fe/Ni surface from a reactive electron donor into l-His-functionalized adsorption platform. This modification promotes surface coordination of Fe/Ni centers with dye functional groups (–SO_3_^−^, –N=N–, –OH), thereby favoring adsorption over catalytic degradation, in contrast to most Fe/Ni systems^46,68^ that predominantly follow catalytic reduction pathways.

### Regeneration and reusability

The easy regeneration and reuse of adsorbents without significant loss of adsorptive capacity are crucial from industrial or practical aspects, making their application more economic and minimizing waste production in wastewater treatment practices.^46^ So, the recovery/desorption of model dyes from spent His@nZVINs was investigated in three different eluents (9 : 1 methanol–acetic acid mixture, 1 M HCl, and 1 M NaOH) to first identify the optimum desorbing media, and related findings are given in Table 5 with empirical conditions. The highest percent dye desorption of 62–64% was achieved in NaOH, with clear pink (E123) or blue-purple (E151) color of dye in the solution. It occurs most likely due to neutralization of positive surface charge of His@nZVINs at basic pH, reducing coulombic interaction among anionic dyes and His@nZVINs, thereby promoting them to desorb in a basic medium. Consensually, owing to strong electrostatic dye–His@nZVINs attraction in the acidic medium, the HCl solution remained inappropriate for dye desorption (Table 5); rather, it showed dissolution of His@nZVINs, evident from brownish color in solution.^37^ The significant dye desorption in an alkaline environment as opposed to none at all under acidic media is in full coherence with pH study findings. In polar organic solvent mixture CH_3_OH–CH_3_COOH (MeOH–AcOH), the small desorption of E127 (3%) and E151 (1%) may be attributed to minor dissolution of dyes, facilitated by their polar organic nature. Thus, about 36–38% dye in NaOH while 97–99% dye in MeOH–AcOH remained adsorbed onto His@nZVINs for the initial desorption run, probably due to strong hydrophobic and hydrogen bond dye–His@nZVINs interactions, working in competition with other stated interactions. The idea of hydrophobic/hydrogen contacts is also strengthened by appreciable dye adsorption at adsorbent pH_PZC_.

**Table 5:** Desorption of E123 and E151 dyes from used His@nZVINs in three eluents

S. no.	Eluent	% Desorption E123^a^	% Desorption E151^a^
1	NaOH (1 M)	64.0	62.1
2	CH_3_OH : CH_3_COOH (9 : 1)	3.0	1.2
3	HCl (1 M)	0.0	0.0

a Empirical conditions: temp. = 298 K, adsorbent amount 0.5 g L^−1^, exposure time = 65 min.

**Fig. 12:**
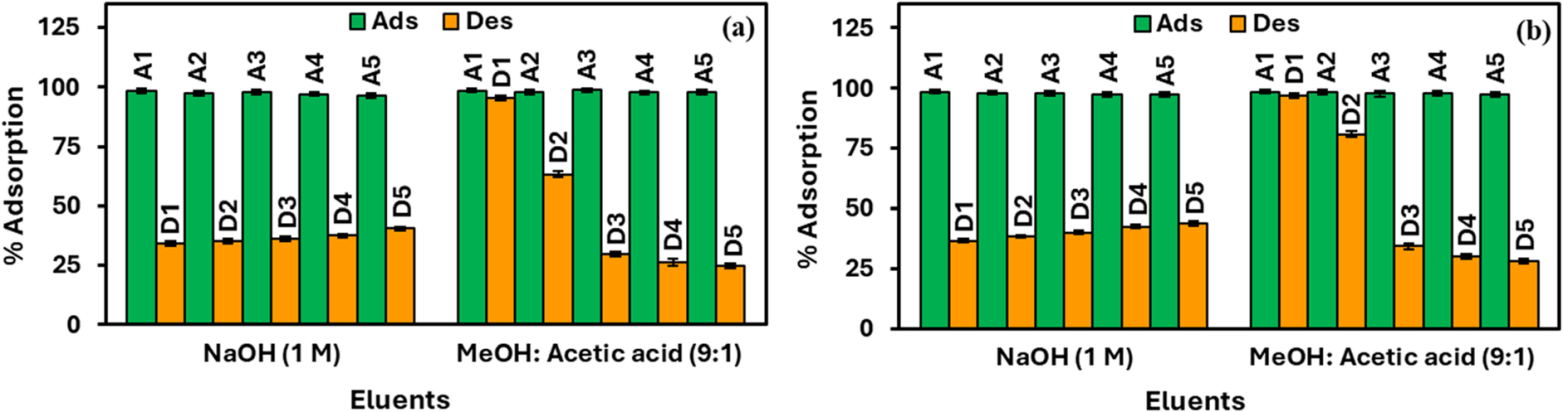
Reusability of His@nZVINs as an adsorbent of (a) E123 and (b) E151. A1–A5 and D1–D5 respectively symbolize successive adsorption (at pH 4) and desorption (in 1 M NaOH or a 9 : 1 methanol–acetic acid mixture) for five cycles [other conditions: initial dye conc. = 20 µM, temp. = 298 K, adsorbent amount = 0.5 g L^−1^, and contact time = 65 min].

Considering the above results, NaOH and MeOH–AcOH were selected for more comprehensive analysis of His@nZVINs reusability. Purposely, the restored His@nZVINs were redeployed up to five successive adsorption–desorption cycles. The outcomes of the reusability test are displayed in Fig. 12a for E123 and Fig. 12b for E151. Compared to the first cycle, the % dye desorption from His@nZVINs in NaOH was gradually decreased up to the fifth cycle, with a difference of 5.4% for E123 and 7.4% for E151. However, the percent adsorption on His@nZVINs remained almost the same and quite high in five consecutive cycles for E123 (99.7–98.5%) and E151 (99.9–98.5%) in the case of recovery from NaOH. Unlike NaOH, MeOH–AcOH interestingly demonstrated a noticeable increase in the capability of used His@nZVINs to desorb anionic dyes toward the next cycles, rising desorption up to 73% for E123 and 70% for E151 in the fifth cycle. This is expected due to better overcoming the strong intermolecular dye–adsorbent hydrophobic/hydrogen linkages by organic solvents, after having a softening effect on the His@nZVINs surface and inside the edifice caused by multiple washings following initial use and hence favoring desorption in successive runs.^73^ The adsorption efficiency of His@nZVINs in the case of MeOH–AcOH was well maintained until the 5th adsorption run with values ≥99.4% for both dyes. The maximum equilibrium time of desorption of the two dyes was 65 min in MeOH–AcOH while it was 40 min in NaOH, indicating their fast and facile desorption.

The in-depth analysis proves that both NaOH and MeOH : AcOH (9 : 1) are effective desorbing agents of anionic dyes to regenerate His@nZVINs, providing them with excellent reusability as an adsorbent. Furthermore, NaOH and MeOH–AcOH serve under different mechanisms to desorb dyes from spent His@nZVINs, predominantly by overcoming the intermolecular (adsorbent–dye) electrostatic barrier and hydrophobic/hydrogen linkages, respectively. This further recommends the combined use of NaOH and MeOH–AcOH as a single desorbing system that would enhance regeneration *via* cumulative/synergistic effects by simultaneous disruption of electrostatic and other non-covalent interactions, reducing dye retention (enhanced desorption), minimizing pore blockage, restoring adsorption capacity, and thereby improving the long-term reusability of His@nZVINs.

### Toxicity study

To assess the environmental biocompatibility of His@nZVINs, two cytotoxicity assays were employed: an *in vitro* MTT assay^60^ using two cell lines, 3T3 (healthy mouse fibroblasts) and HeLa (human cervical cancer cells), and the brine shrimp lethality assay (BSLA).^61^ The cytotoxicity results of His@nZVINs are summarized in Table 6 as percent cell growth inhibition (MTT assay at 30 µg mL^−1^) and percent mortality (BSLA at 1, 10, and 100 µg mL^−1^) and are compared with those of precursors (bare nZVINs and free l-His) and standard cytotoxic drugs (doxorubicin for MTT and etoposide for BSLA).

**Table 6:** Toxicity assessment of His@nZVINs, in comparison with precursors (nZVINs and l-His)

Compounds	MTT cytotoxicity assay (% inhibition)		Brine shrimp lethality assay (% mortality)^a^		
	3T3 (normal fibroblasts)	HeLa (cervical cancer cells)	*Artemia salina* (nauplii)		
	At 30 µg mL^−1^	At 30 µg mL^−1^	At 1 µg mL^−1^	At 10 µg mL^−1^	At 100 µg mL^−1^
His@nZVINs	13.5	0.0	3.3	10.0	20.0
nZVINs	0.7	9.9	13.3	13.3	20.0
l-His	30.8	0.0	10.0	13.3	13.3
Standard drugs	—	—	—	—	—
Doxorubicin	89.9	100.0	—	—	—
Etoposide	—	—	—b	—	—

a Brine shrimp (nauplii) count at the start = 30.

b Etoposide was evaluated only at 7.5 µg mL^−1^ (brine shrimp percent mortality = 70.0%).

His@nZVINs showed no detectable growth inhibition of HeLa cells and only minor inhibition of 3T3 cells (13.5%), compared with doxorubicin that possessed substantially higher cytotoxicity, causing 89.9% inhibition of 3T3 and complete (100%) inhibition of HeLa cells at the same dose. Similarly, in the BSLA, His@nZVINs induced low mortality (3–20%) across the tested concentration range (1–100 µg mL^−1^), whereas etoposide caused 70% mortality at 30 µg mL^−1^. These results indicate that His@nZVINs exhibit negligible cytotoxicity under the tested conditions. Comparison with the individual precursors revealed that His@nZVINs are less cytotoxic toward 3T3 cells (13.5% inhibition) than l-His (30.8% 3T3 inhibition) while being noncytotoxic toward HeLa cells relative to bare nZVINs (9.9% HeLa inhibition). Consistently, in the BSLA at 1–10 µg mL^−1^, His@nZVINs produced lower mortality (3–10%) than both l-His (10–13% mortality) and bare nZVINs (13% mortality). Moreover, the observed cytotoxicity of His@nZVINs was lower than the cumulative (additive) cytotoxic effect of the individual precursors across all test systems (3T3, HeLa, and brine shrimp). These findings demonstrate a synergistic reduction in inherent cytotoxicity upon the integration of nZVINs and l-His into His@nZVINs, resulting in improved overall biocompatibility.

Cytotoxicity data for nZVIN-based BNPs are not available in the literature for direct comparison. However, several other nanoparticles, such as Au/Ag BNPs,^77^ TiO_2_,^78^ plant-mediated iron oxides,^79^ CuO,^78^ and AgNPs,^61^ previously reported with low-toxicity in the BSLA, exhibit greater lethality toward brine shrimp than His@nZVINs at comparable concentrations and exposure times. This suggests His@nZVINs as a safe adsorbent with minimal cytotoxic risk to marine and terrestrial life.

## Conclusions

This study presents the successful synergistic design of core–shell l-His-functionalized Fe/Ni nanospheres (His@nZVINs) for efficient adsorptive removal of hazardous anionic food dyes E123 and E151 from water. His@nZVINs combine non-cytotoxicity, stability, switchable-charge, and multi-interacting sites, enabled by l-His anchoring *via* carboxyl and imidazole moieties. The zwitterionic His@nZVINs afforded high removal efficiencies (97.6% for E123; 99.4% for E151) at native pH, increasing to ∼100% at pH 4 (20 µM dye, 0.5 g per L adsorbent). Adsorption equilibrium was attained rapidly within 15–120 min for E123 and 20–150 min for E151 under varied temperature, adsorbent dose, pH, and salt/dye conditions. The relatively higher removal selectivity of His@nZVINs for E151 was likely driven by its greater negative charge, polar functionalities, and aromatic hydrophobicity, whereas the faster uptake of E123 was attributable to its smaller molecular size. His@nZVINs adsorption predominantly followed pseudo-2nd order kinetics, film diffusion, monolayer surface coverage, and an exothermic physisorptive mechanism. His@nZVINs retained high performance in electrolyte-rich and real water matrices and were readily regenerated using NaOH and MeOH–AcOH, demonstrating excellent reusability and practical applicability. Consistent with FT-IR evidence and desorption behavior, dye uptake was governed by synergistic electrostatic, hydrogen-bonding, metal–dye, and π–π interactions. Given their facile synthesis, superior *q*_max_ values (122.0 mg g^−1^ for E123; 131.6 mg g^−1^ for E151), rapid uptake, eco-friendliness, magnetic separability, structural selectivity, and reusability, His@nZVINs emerge as a viable, economical, and efficient alternate platform for anionic dye remediation in water, warranting further evaluation in multicomponent pollutant systems and real industrial effluents.

## Author contributions

Q-A: conceptualization, methodology, data curation, formal analysis & validation, visualization, writing – original draft, writing – review & editing, funding acquisition, project administration, resources. AS: data collection, formal analysis, partial writing – original draft. MRS: conceptualization, supervision, writing – review, AFM data validation, provision of material organic coating. MIM: thermal gravimetric data collection & validation.

## Conflicts of interest

The authors declare no competing interests.

## Supplementary Material

NA-OLF-D5NA01180A-s001

## Data Availability

The information supporting the manuscript (synthesis scheme, real photographs depicting dye removal by His@nZVINs, isothermal/kinetic model linear expressions and parameters, EDX spectrum and elemental maps of bare nZVINs, FT-IR spectra of free E123/E151 dyes, and basic water chemistry parameters and ionic composition of investigated real water matrices) is available as part of the supplementary information (SI). The references cited in the SI are already part of the article's reference list. Supplementary information is available. See DOI: https://doi.org/10.1039/d5na01180a.
