# Supplementary material for: Ecofriendly amino acid-tailored Fe/Ni nanomagnets for efficient adsorptive removal of food azo dyes (E123/E151) via electrostatic–hydrophobic contacts
Source: Nanoscale Adv. 2026 Aug 4;8(17):4870–93. doi: 10.1039/d5na01180a (PMC13435052; doi:10.1039/d5na01180a)
Supplement: Supplementary file 1 [file NA-008-D5NA01180A-s001.pdf]

Supplementary Information (SI) for Nanoscale Advances.
This journal is © The Royal Society of Chemistry 2026

ELECTRONIC SUPPLEMENTARY INFORMATION

Ecofriendly amino acid-tailored Fe/Ni nanomagnets for efficient adsorptive removal of food azo dyes (E123/E151) via electrostatic-hydrophobic contacts

Qurrat-ul-Ain,*[a] Abira Saleem,[a] Muhammad Raza Shah [b] and Muhammad Imran Malik [b]

[a] *Department of Chemistry, University of Karachi, Karachi 75270, Pakistan.*
[b] *H.E.J. Research Institute of Chemistry, International Center for Chemical and Biological Sciences, University of Karachi, Karachi 75270, Pakistan.*

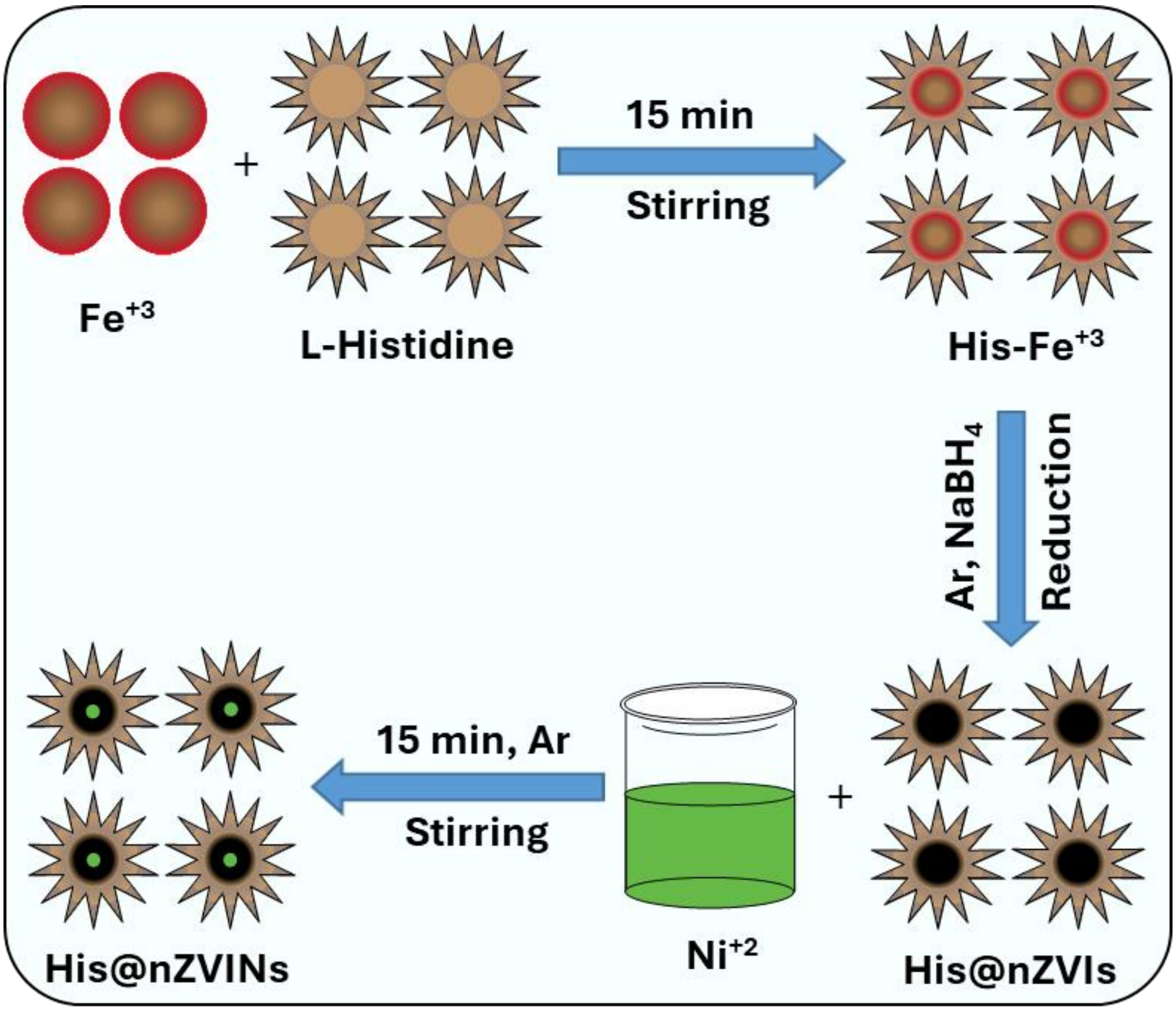

Scheme S1 Synthesis of **His@nZVINs**.

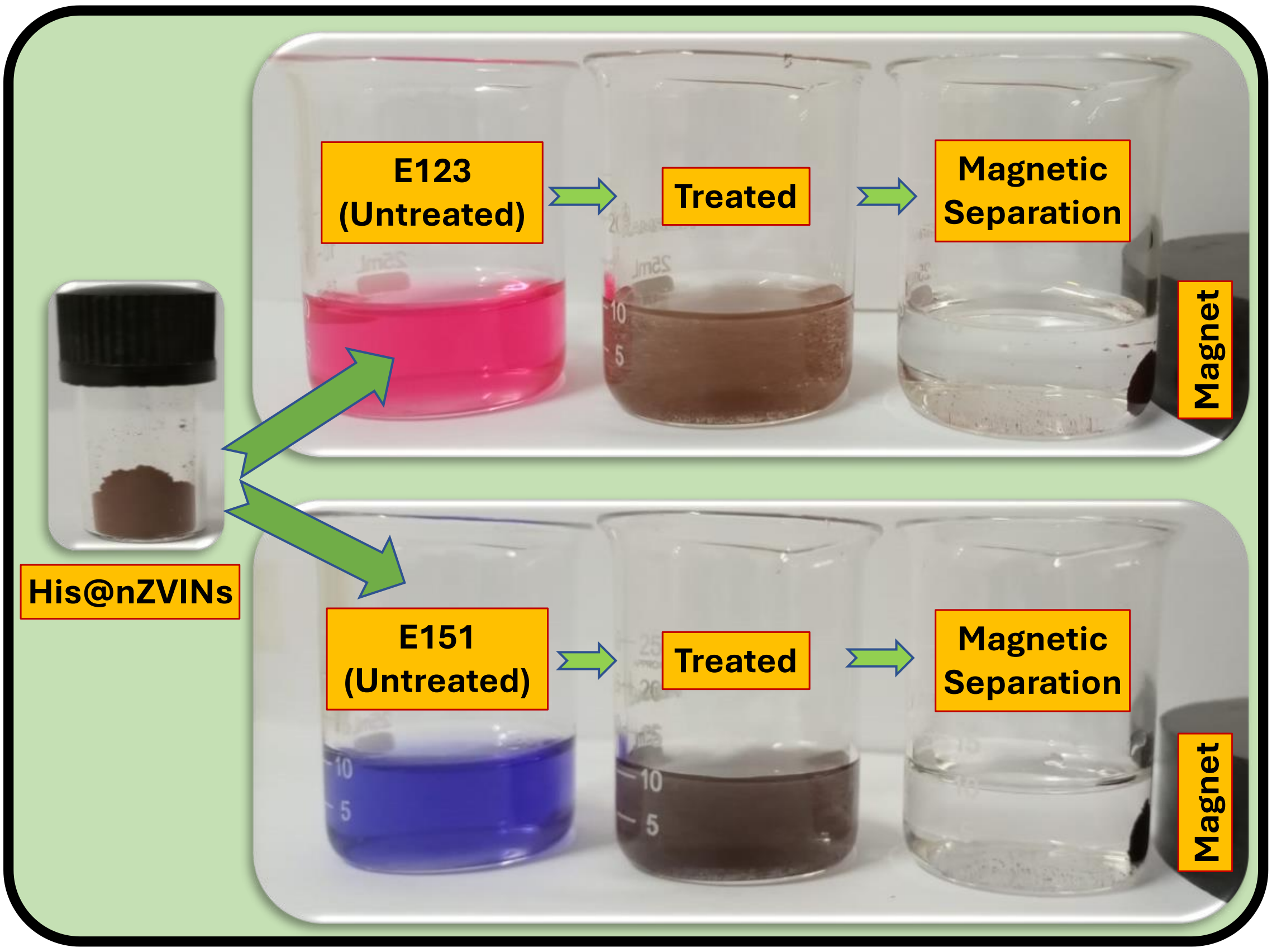

Fig. S1 Some real photographs depicting efficient elimination of food azo dyes (E123 and E151) from water using **His@nZVINs** adsorbent, followed by magnetic separation.

Adsorption isotherms: Mathematical linear expressions

Four isothermal models applied for current study are represented by respective straight-line equations as given in Table S1.

Table S1 Adsorption isotherms with linear expressions and plotting parameters

Isothermal model	Linear expression	Plot	References
Langmuir	$C_e/q_e = 1/(q_{max}K_L) + (1/q_{max})C_e$	C_e/q_e vs. C_e	1
Freundlich	$\log q_e = \log K_F + (1/n)\log C_e$	$\log q_e$ vs. $\log C_e$	2
Dubinin-Radushkevich	$\ln q_e = \ln q_m - K_{DR}€^2$	$\ln q_e$ vs. $€^2$	3
Temkin	$q_e = (RT/b)\ln A + (RT/b)\ln Ce$	q_e vs. $\ln C_e$	4

In the expression of Langmuir isotherm, the variables C_e, q_e, K_L, and q_{max} correspond to the dye solution concentration at equilibrium (measured in mg L^{-1}), the amount of dye loaded onto the adsorbent surface at equilibrium (measured in mg g^{-1}), the constant associated with the adsorption free energy (measured in L mg^{-1}), and adsorbent's maximum adsorptive capacity for an entire monolayer coverage (mg g^{-1}).[5,6] For the linear expression of Freundlich isotherm, the symbol K_F represents the Freundlich constant ($mg^{1-1/n}$ $L^{1/n}$ g^{-1}), describing the adsorbent's relative adsorptive capacity. Another related dimensionless constant 'n' functions as a heterogeneity factor and indicates the strength or intensity of adsorption.[4] The Dubinin-Radushkevich (D–R) isothermal constant (measured in mol^2 J^{-2}) is denoted by K_{DR}, whereas q_m stands for the predicted saturation adsorption capacity (measured in mg g^{-1}).[3] In the Temkin model, A represents the binding constant of equilibrium, which reflects the maximal binding energy and is expressed in terms of L g^{-1}. The adsorption heat corresponds to the Temkin constant b, which is expressed in J mol^{-1}. R in Temkin expression stands for the universal gas constant, which has a numerical value of 8.314 J $mol^{-1}K^{-1}$, and T represents the absolute temperature measured in Kelvin.[2]

Adsorption Kinetics models: Mathematical linear expressions

The adsorption rate from the pseudo-1st order (Lagergren's) model and the pseudo-2nd order model can be ascertained by the linearized integral equations, eqn (S1) and eqn (S2), respectively.[4]

$$\ln(q_e - q_t) = \ln q_e - k_1 t \quad \text{(S1)}$$

$$t/q_t = 1/(k_2 q_e^2) + t/q_e \quad \text{(S2)}$$

Wherein, q_e and q_t correspond the quantity of dye adsorbed (mg g^{-1}) onto adsorbent at equilibrium and at time interval t (min). The pseudo-first order and pseudo-second order models' rate constants are represented by the symbols k_1 (min^{-1}) and k_2 (g mg^{-1} min^{-1}), correspondingly. The rate constants (k_1 and k_2), equilibrium adsorptive capacity (q_e), and correlation coefficients (R^2) for dyes adsorption are estimated through the slopes and intercepts of linear plots of pseudo-1st order model [ln($q_e - q_t$) versus t] and pseudo-2nd order model [t/q_t versus t]. The linear integral rate equation of Elovich kinetics model is expressed as eqn (S3).[7]

$$q_t = (1/b)\ln(ab) + (1/b)\ln t \quad \text{(S3)}$$

The variable a (mg g^{-1} min^{-1}) in Elovich equation represents the initial rate of adsorption, while b (g mg^{-1}) denotes the desorption constant associated with the amount of surface covering. The Elovich coefficients a and b values are computed through the intercept and slope of the linear plot between q_t and ln t. According to intra-particle diffusion model, the adsorption capacity (q_t) at a time 't' varies almost proportionally with $t^{1/2}$, as shown in eqn (S4).[8]

$$q_t = k_{id}\, t^{1/2} + C_i \quad \text{(S4)}$$

Where C_i is a parameter that represents the boundary layer's thickness (mg g^{-1}), and K_{id} is the intraparticle diffusion rate constant (mg g^{-1} $min^{-1/2}$). The values of K_{id} and C_i for dye adsorption onto adsorbent are estimated through the slope and intercept of the curve of q_t against $t^{1/2}$.[9] Boyd model plot is drawn between B_t and contact time (t). The B_t is calculated using eqn (S5).[6]

$$B_t = -0.4977 - \ln(1 - F) \quad \text{(S5)}$$

Where B_t depends on F, and F represents the fraction of dye deposited on adsorbent at different time intervals t (min), calculated as F = q_t/q_e.[3]

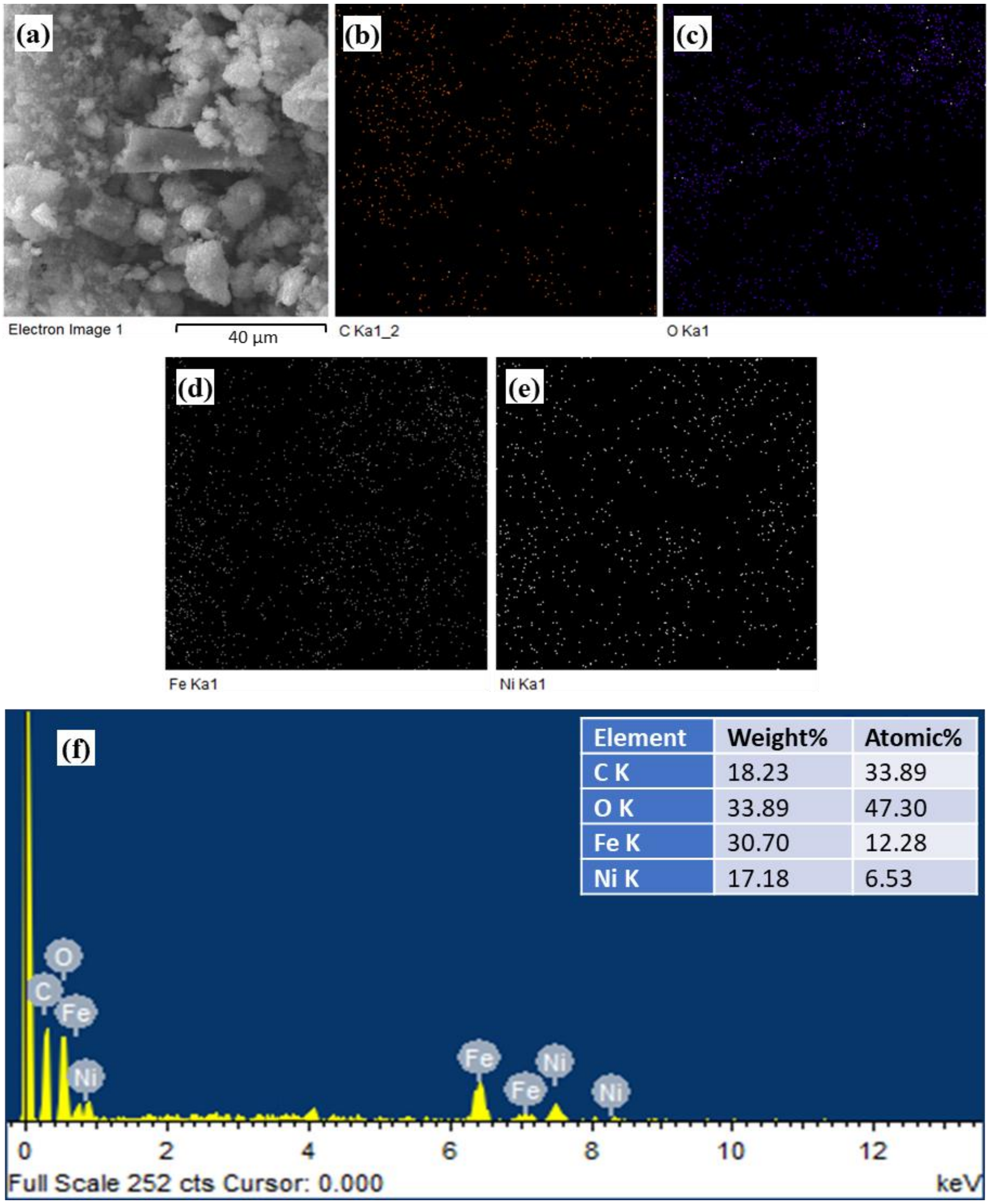

Fig. S2 EDS analysis of bare nZVINs. (a) SEM microstructure of selected mapping area. (b–e) Corresponding EDS elemental mapping of C, O, Fe, and Ni, respectively. (f) EDX spectrum with elemental composition.

Table S2 Basic water chemistry parameters and major ionic composition of the investigated real water samples

Water type	pH	TDS (ppm)	EC (µs cm^{-1})	Salinity (ppm)	Na^+ (ppm)	K^+ (ppm)	Ca^{2+} (ppm)	Mg^{2+} (ppm)	Cl^- (ppm)	HCO_3^- (ppm)	NO_3^- (ppm)	SO_4^{2-} (ppm)
Drinking water	6.10	160	322	168	18	2	8	1	53	5	0.4	13
Tap water	7.30	403	813	410	75	7	40	6	62	80	0.5	90
Groundwater	7.53	3500	6217	3450	1010	8	120	117	1596	412	20	210
Seawater	7.56	26500	53009	38420	8406	176	349	797	15957	94	2	1519

TDS = total dissolved solids; EC = electrical conductivity.

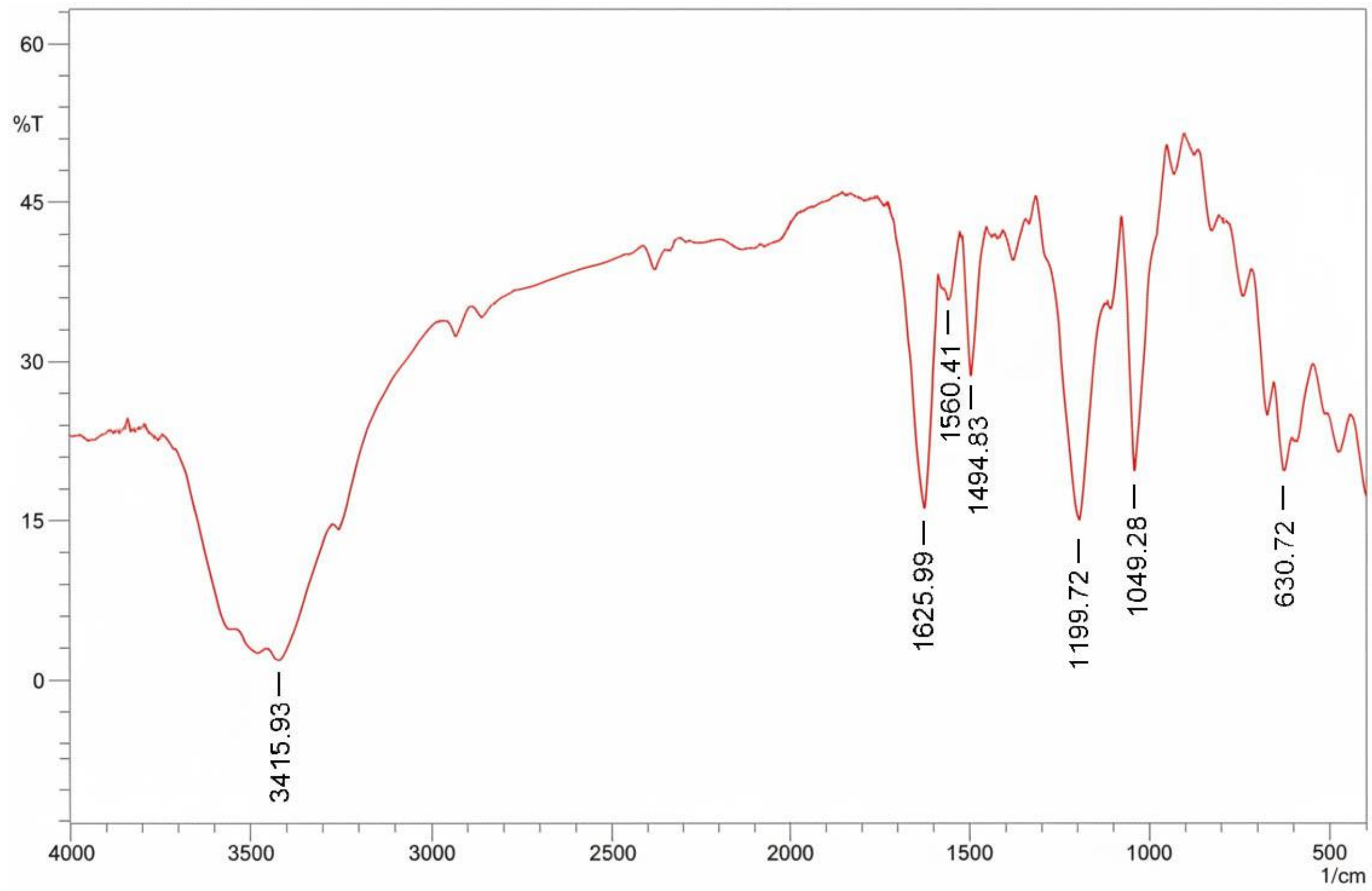

Fig. S3 FT-IR spectrum of E123 dye.

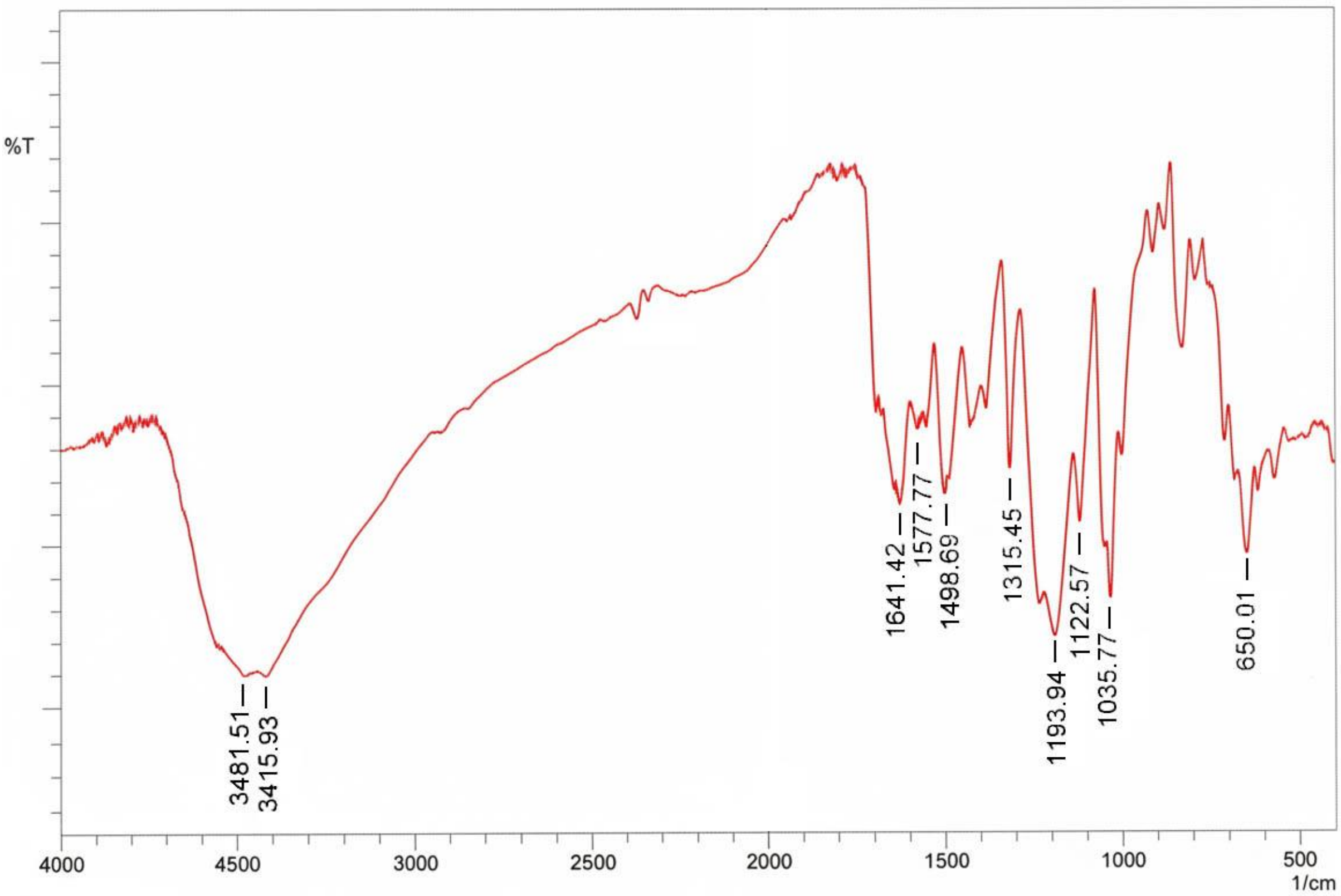

Fig. S4 FT-IR spectrum of E151 dye.

References

1 M Sofuoglu, A. E. Kuyucu, K. Erol and F. Gokmese, *Gels*, 2025, **11**, 411.
2 Qurrat-ul-Ain, J. Khatoon, M. R. Shah, M. I. Malik, I. A. T. Khan, S. Khurshid and R. Naz, *RSC Adv.*, 2019, **9**, 2978–2996.
3 S. Banerjee and M. C. Chattopadhyaya, *Arab. J. Chem.*, 2017, **10**, S1629–S1638.
4 M. N. Zafar, Q. Dar, F. Nawaz, M. N. Zafar, M. Iqbal and M. F. Nazar, *J. Mater. Res. Technol.*, 2019, **8**, 713–725.
5 S. Eftekhari, M. R. Sohrabi and S. Mortazavinik, *Iran. J. Chem. Chem. Eng.*, 2024, **43**, 2945–2958.
6 K. O. Kazimirova and S. N. Shtykov, *J. Anal. Chem.*, 2024, **79**, 1724–1732.
7 L. Natrayan, S. A. Kalam, S. Sheela, P. Paramasivam and K. Shanmugam, *Discov. Appl. Sci.*, 2024, **6**, 662.
8 M. Munir, M. F. Nazar and M. N. Zafar, *Int. J. Environ. Anal. Chem.*, 2021, **101**, 2848–2865.
9 A.-N. M. Salem, M. A. Ahmed and M. F. El-Shahat, *J. Mol. Liq.*, 2016, **219**, 780–788.
